# Supplementary material for: Soft ferroelectret ultrasound receiver for targeted peripheral neuromodulation
Source: Nat Commun. 2023 Dec 16;14:8386. doi: 10.1038/s41467-023-44065-6 (PMC10725454; doi:10.1038/s41467-023-44065-6)
Supplement: Supplementary file 1 — Supplementary Materials [file 41467_2023_44065_MOESM1_ESM.docx]

Supplementary Materials for

**Soft Ferroelectret Ultrasound Receiver for Targeted Peripheral Neuromodulation**

Tong Li^1,2,3,†^, Zhidong Wei^1,†^, Fei Jin^1,†^, Yongjiu Yuan^2^, Weiying Zheng^1^, Lili Qian^1^, Hongbo Wang^2^, Lisha Hua^2,4^, Juan Ma^1^, Huanhuan Zhang^2^, Huaduo Gu^2^, Michael G. Irwin^4^, Ting Wang^5,^*, Steven Wang^2,3,^*, Zuankai Wang^6,^*, Zhang-Qi Feng^1,^*

^1^ School of Chemistry and Chemical Engineering, Nanjing University of Science and Technology, Nanjing 210094, China

^2^ Department of Mechanical Engineering, City University of Hong Kong, Hong Kong 999077, China

^3^ Research Center for Nature-inspired Engineering, City University of Hong Kong, Hong Kong 999077, China

^4^ Department of Anaesthesiology, The University of Hong Kong, Hong Kong 999077, China

^5^ State Key Laboratory of Bioelectronics, Southeast University, Nanjing 210096, China

^6^ Department of Mechanical Engineering, The Hong Kong Polytechnic University, Hong Kong 999077, China

^†^These authors contributed equally to this work

*Corresponding author. Email: tingwang@seu.edu.cn, steven.wang@cityu.edu.hk, zk.wang@polyu.edu.hk, fengzhangqi1981@163.com

This PDF file includes:

Supplementary Figs. 1 to 29

Supplementary Tables 1 to 3

Supplementary References 1 to 70

**Supplementary Figures**


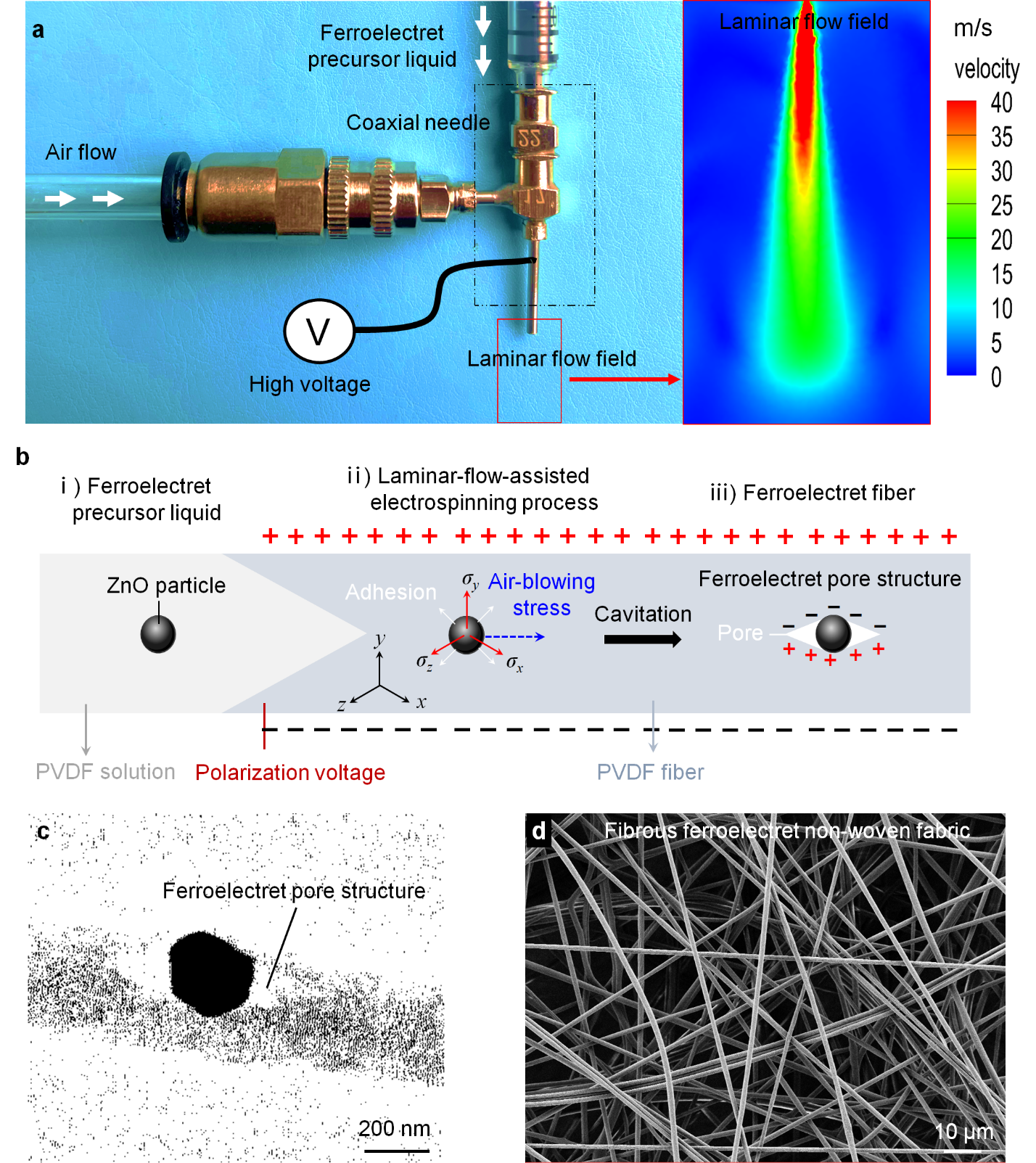


**Supplementary Fig. 1.** **Preparation process of ferroelectrets.** **a,** Laminar flow field formed during the electrospinning process. **b,** Schematic showing the cavitation effect of ZnO particles and PVDF matrix under air-blown stretch stress. **c,** Sharpened TEM image clearly showing the existence of the ferroelectret pore structure. **d,** SEM image of multiple ferroelectret fiber-based non-woven fabric of uniform size.

In the presence of particles, cavitation will be induced by the interfacial debonding of a filler from the matrix.^1^ Cavitation by debonding can be facilitated by the weak adhesion of filler particles to the matrix. In fact, during the deformation process of semicrystalline nanocomposites, nanoparticles act as stress concentrators and the stress concentration gives rise to a build up of triaxial stress in the vicinity of the particles (Supplementary Fig. 1b). The void formation takes place via a debonding process at the matrix/nanoparticles interface, when the stress concentration reaches a critical value, which may constitute the stress at yield point. The debonding appears on both sides of the particles, parallel to the direction of applied stress, and increases with increasing strain. Also, when the composite is subjected to stress, the probability of void formation increases, as the difference in Young's modulus between the polymer and nanofiller is usually significant.^2^ As confirmed, when the blowing tensile stress acts on the ferroelectret precursor, the cavitation effect occurs on both sides of the ZnO particle and parallel to the blowing direction (Supplementary Fig. 1c). In contrast, the PVDF/ZnO composite fibers had no cavitation at all (Supplementary Fig. 2). Consistent with previous findings, tensile strain at the onset of cavitation can serve as a determinant of adhesion strength between the polymer matrix and nanofiller grains.^3^ Finally, we successfully prepared fibrous ferroelectret non-woven fabric composed of PVDF and ZnO using the laminar-flow-assisted electrospinning method (Supplementary Fig. 1d).


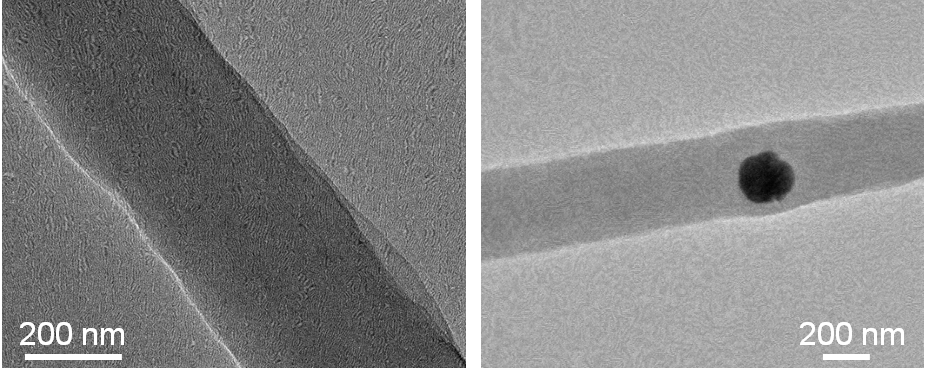


**Supplementary Fig. 2.** **TEM images of a single PVDF fiber and PVDF/ZnO composite fiber.** In the PVDF/ZnO fiber, no void was found at the interface of ZnO particle and PVDF matrix without air blowing. n = 3 independent experiments showing similar results.


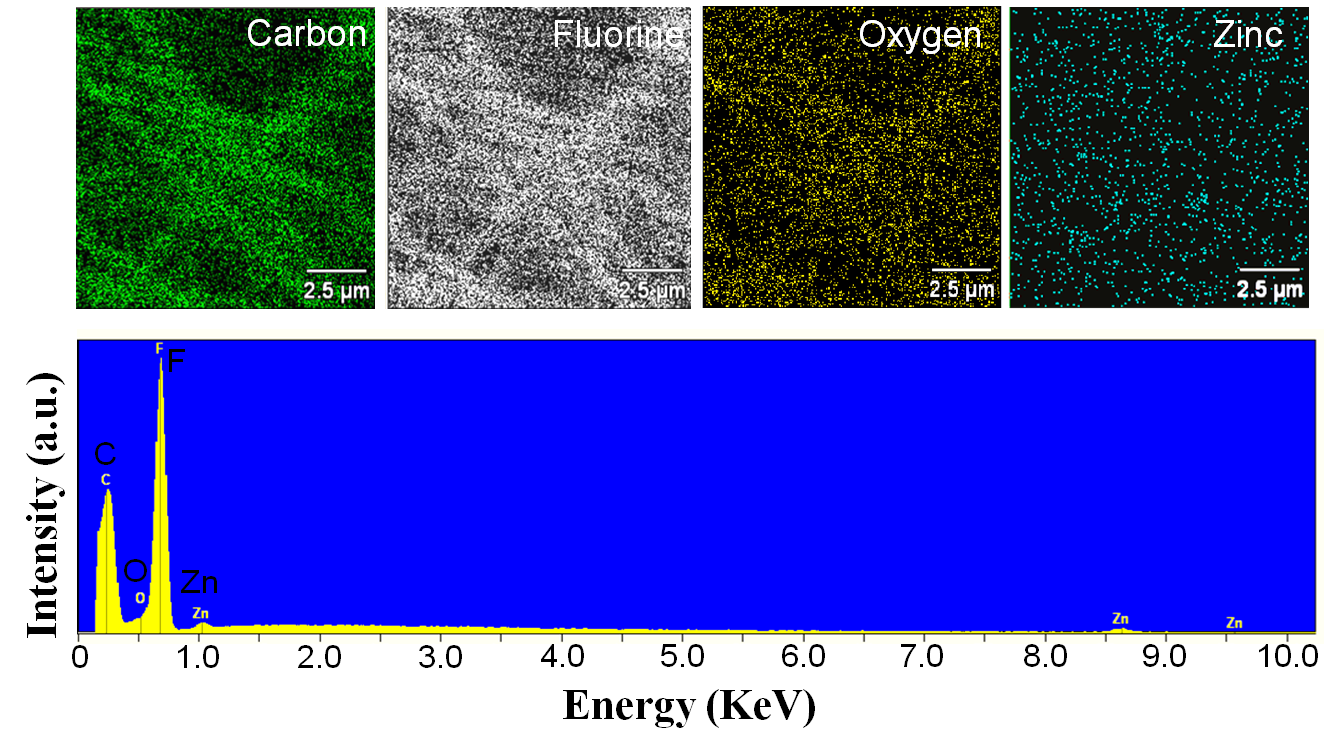


**Supplementary Fig. 3.** **Elemental analysis of ferroelectrets.** Energy dispersive spectrometer mapping and its corresponding profiles of ferroelectret fibers. n = 3 independent experiments showing similar results.


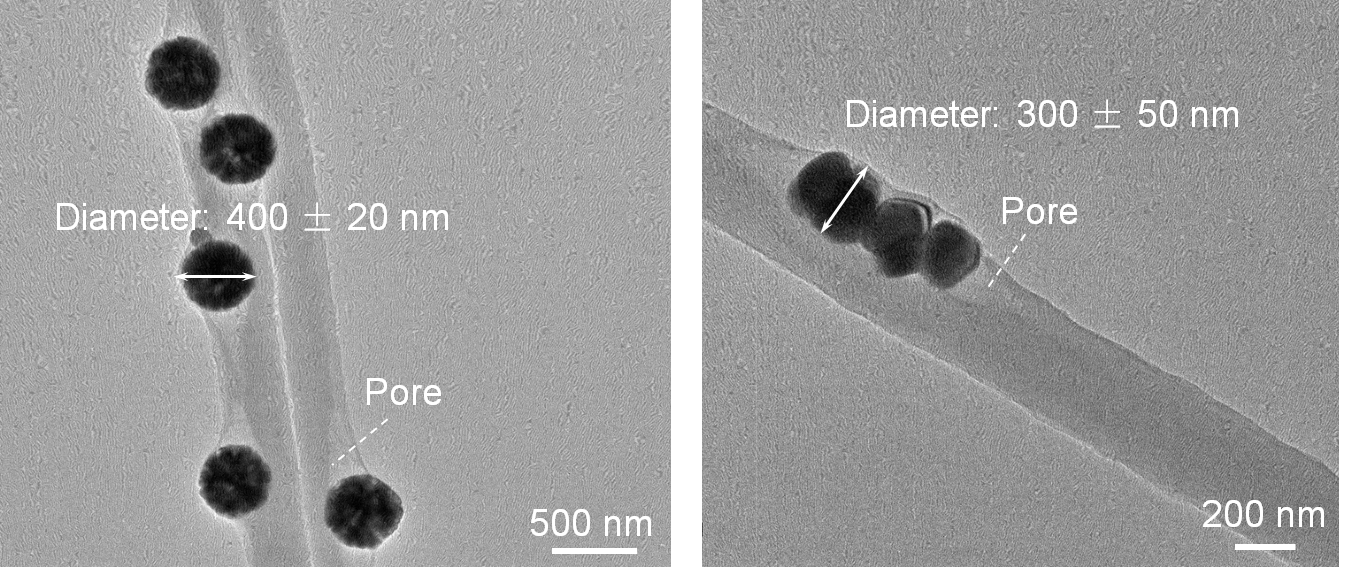


**Supplementary Fig. 4.** **Characterization of ferroelectret pore structure.** TEM image revealing the ferroelectret pore structure existing between ZnO and PVDF of different sizes. Even when the ZnO particles are agglomerated, the ferroelectret pore structure can still be produced. n = 3 independent experiments showing similar results.


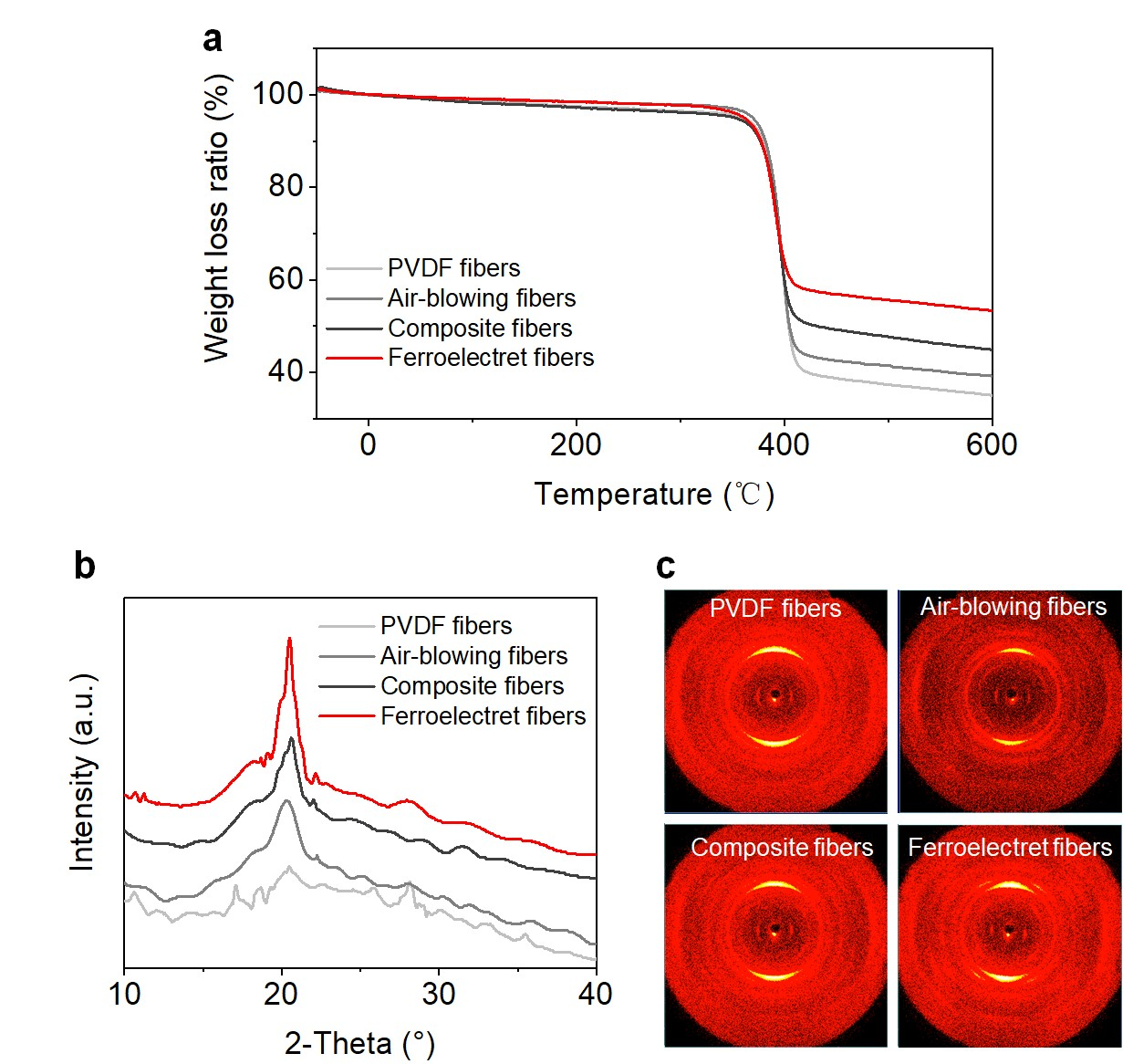


**Supplementary Fig. 5. Crystallinity characterization of ferroelectrets.** Thermogravimetric analysis curves **(a)** and 1D XRD spectra **(b)** of PVDF fibers, air-blowing fibers, PVDF/ZnO composite fibers, and ferroelectret fibers. **c,** 2D XRD images showing PVDF chain's orientation in PVDF fibers, air-blowing fibers, PVDF/ZnO composite fibers, and ferroelectret fibers.


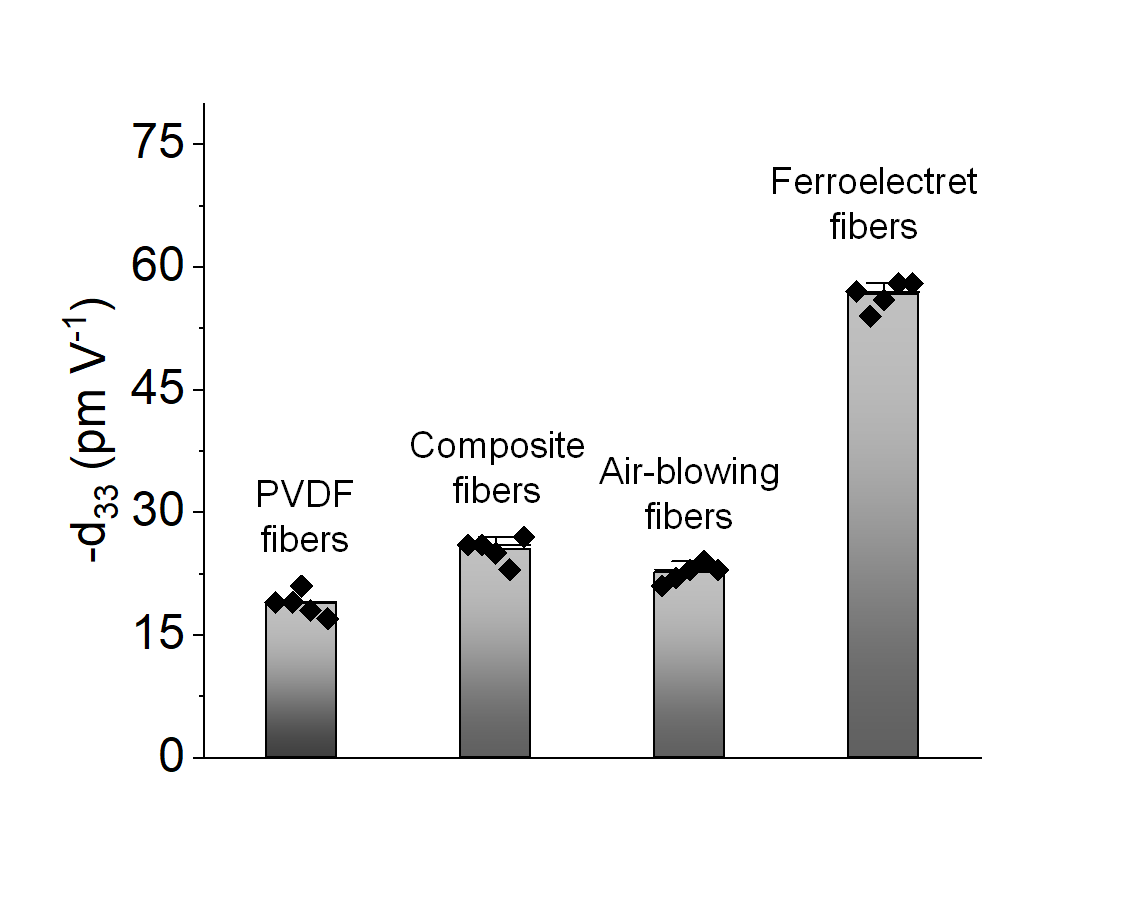


**Supplementary Fig. 6. Characterization of piezoelectric coefficients of ferroelectrets.** Measured d_33_ piezoelectric coefficients of PVDF fibers, PVDF/ZnO composite fibers, air-blowing fibers, and ferroelectret fibers using a quasi-static d_33_ measuring instrument. n = 5 independent samples for each group. Data are expressed as mean values ± SD. All error bars indicate ± SD.

The d_33_ of the ferroelectret fibers is the highest (56 ± 2 pC N^−1^), which is more than twice the air-blowing PVDF fibers (22 ± 2 pC N^−1^) or PVDF/ZnO composite fibers (25 ± 2 pC N^−1^) (Supplementary Fig. 6). To understand this enhancement, we studied the polymer chain’s orientation and crystallinity. First, we performed thermogravimetric analysis on PVDF fibers, air-blowing fibers, PVDF/ZnO composite fibers, and ferroelectret fibers (Supplementary Fig. 5a). All fibers experienced obvious weight loss at 400°C. At this stage, the PVDF molecular chains were decomposed to remove HF in the molecules. Among them, the decomposition rate of ferroelectret fibers is relatively the lowest, indicating its high crystallinity. Crystallinity was further quantified by 1D XRD (Supplementary Fig. 5b). We calculated the crystallinity of the PVDF fibers, air-blowing fibers, PVDF/ZnO composite fibers, and ferroelectret fibers to be 27.5%, 52.1%, 66.3%, and 68.9%, respectively. According to references,^4, 5, 6^ the enhanced d_33_ can be attributed to the existence of the oriented amorphous fraction that exhibits improved dipole mobility and better chain alignment after the air-blowing process, enhancing the piezoelectric properties of the fiber. Therefore, we used 2D XRD to further study the orientation of the polymer chain in the fiber. As shown in Supplementary Fig. 5c, the results reveal that the air-blowing electrospin process can align the polymer chains along the fiber axis direction. The orientation degrees of fibers are quantified using Herman’s orientation factor. We calculated the orientation degrees of the PVDF fibers, air-blowing fibers, PVDF/ZnO composite fibers, and ferroelectret fibers, using Herman’s orientation factor to be 0.78, 0.83, 0.80, and 0.81, respectively. Overall, the comparable crystallinities between the PVDF/ZnO composite fibers and ferroelectret fibers as well as the comparable orientation degrees between the air-blowing fibers and ferroelectret fibers suggest that the enhanced value of the piezoelectric coefficient is driven neither primarily by a flow induced orientation effect nor by the crystallinity, but rather by another mechanism. Therefore, there must be a synergistic effect from both the PVDF matrix and ZnO particles. We thus performed TEM characterization on the air-blowing PVDF/ZnO fiber (Fig. 1d, Supplementary Fig. 4) and observed cavitation on both sides of the ZnO particles, with the cavities elongated axially along the fiber (i.e., gas blowing direction). The existence of cavities in the vicinity of the ZnO particles, which are found only in the air-blowing fibers and not in the general electrospin composite fibers, leads us to suggest that cavitation in the drawn composite fiber is the major contributor.

From this observation, a mechanism of enhanced piezoelectricity due to the dimensional effect for the air-blowing PVDF/ZnO fiber is proposed (Fig. 1c, Supplementary Fig. 1). With initial electrospining, PVDF undergoes solidification and crystallization. Upon air drawing of the crystallized sample, cavitation takes place around the ZnO particles, forming horizontal pores on the two sides. After electric poling during electrospinning process, ferroelectric domains in PVDF are polarized, generating an electret effect. When deforming the poled PVDF/ZnO fiber during the direct piezoelectric test, the pore volume changes, creating a dimensional effect for enhanced piezoelectricity. That is, the change of dipole density by changing the pore volume induces significantly improved piezoelectricity. Similar PVDF foam electret has been reported to show significantly enhanced piezoelectric performance due to the porous structure.^7^ In this sense, the drawing-aligned oriented amorphous fraction and the increased dielectric constant of the composite have a much weaker contribution to the enhanced d_33_.^5, 8^ It should be noted that conventional foamed ferroelectrets are prone to depolarize and lose their piezoelectric properties rapidly.^9^ Notably, as a representative of semicrystalline dipole electrets, PVDF was found to exhibit considerable piezoelectric and charge stability as early as the 1960s and early 1970s.^10, 11^ Therefore, in this study, for the first time, we introduced ferroelectret pore structure by using a one-step laminar-flow-assisted electrospinning method, further increasing the number of electric dipoles in PVDF dipole electrets, and thus developing a new class of stable fibrous PVDF-based ferroelectret devices. This approach paves a novel route towards a new paradigm of high-performance and stable soft ferroelectret transducers.

In conclusion, the high performance of the thermally drawn PVDF/ZnO fiber can be attributed to cavitation between ZnO particles and the PVDF fiber matrix, and the well-aligned orientated amorphous fraction, which further increases the piezoelectric performance.


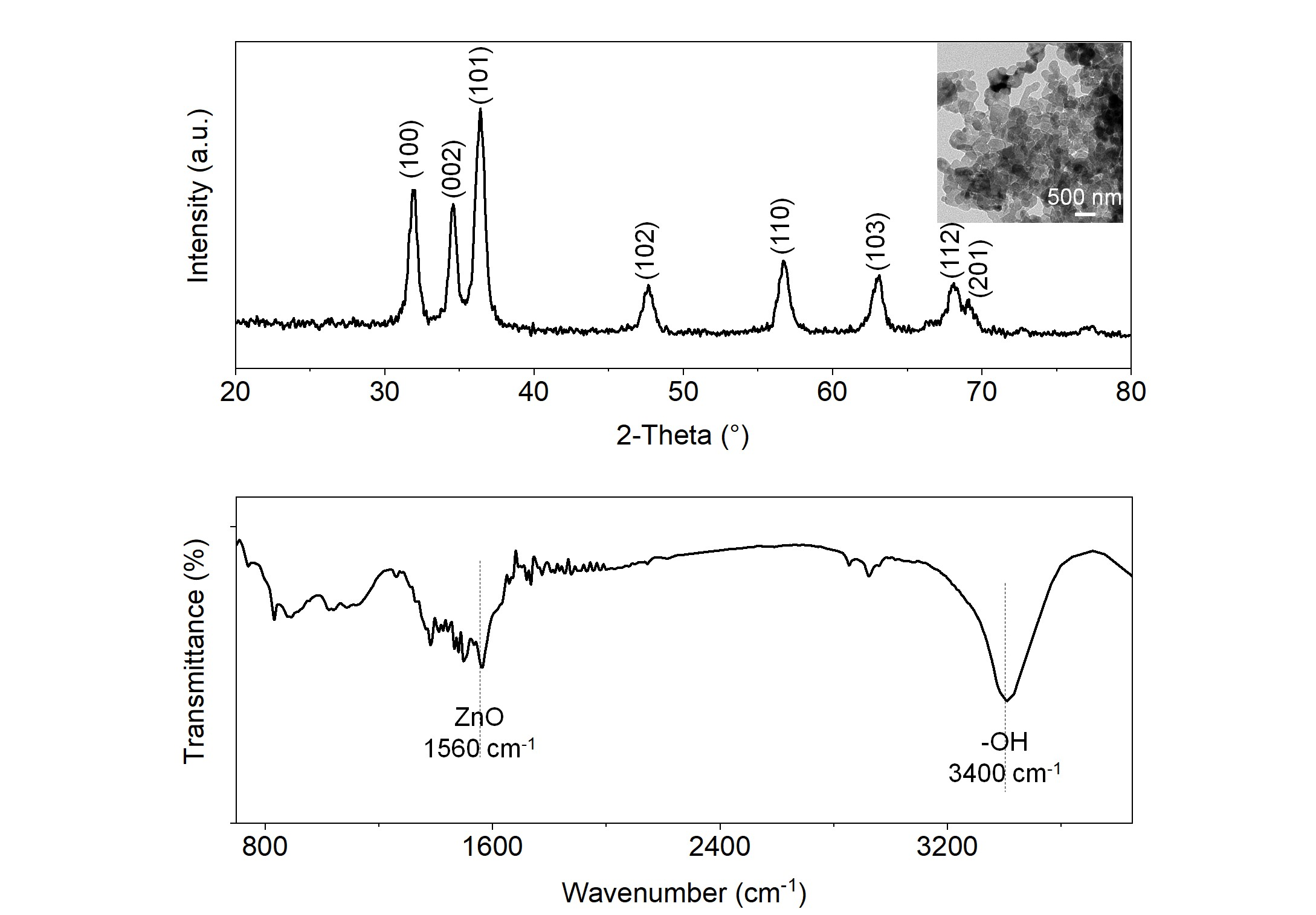


**Supplementary Fig. 7.** **XRD and FTIR patterns of ZnO particles.** Inset: TEM image of ZnO particles.


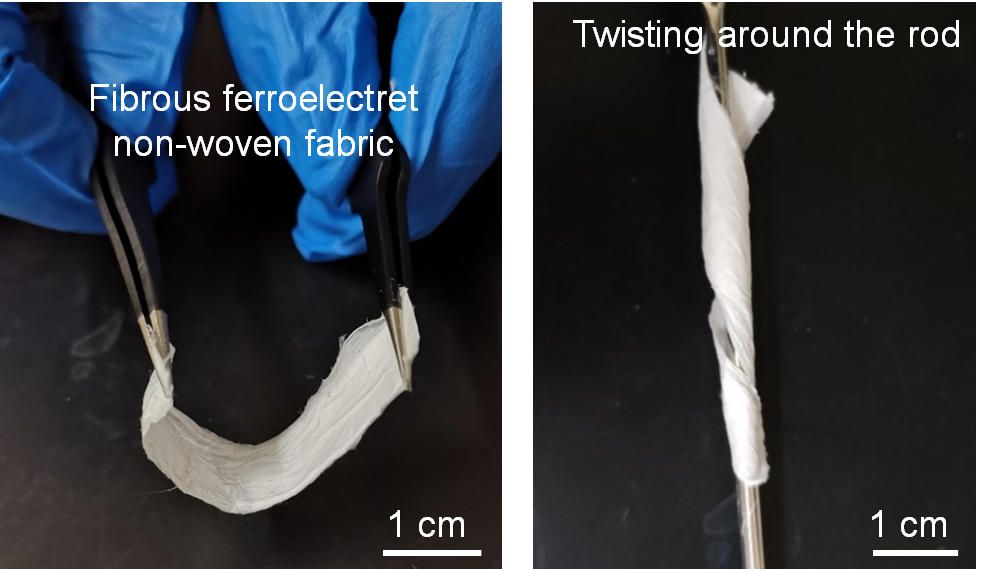


**Supplementary Fig. 8.** **The fibrous ferroelectret non-woven fabric exhibits excellent flexibility and can be easily bent and twisted.**

**
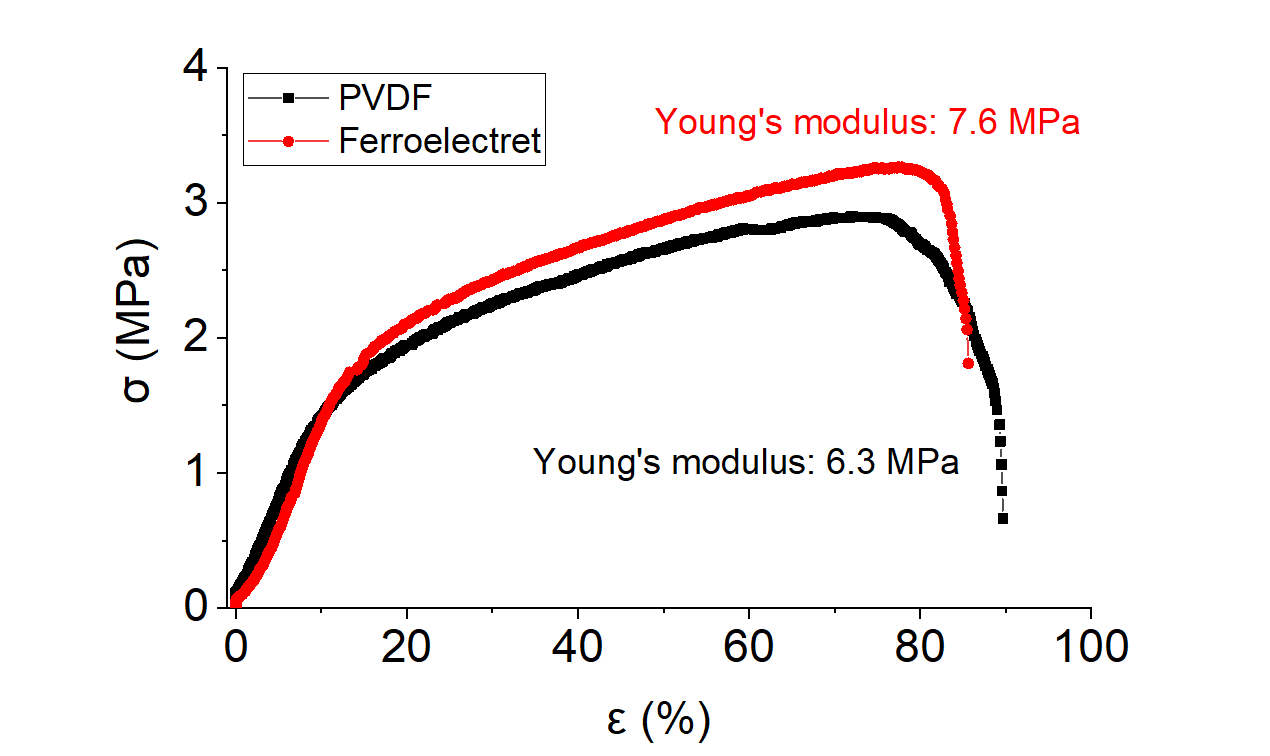
**

**Supplementary Fig. 9.** **Tensile stress-strain curves showing that the tensile strength and strain of the ferroelectret fiber film and pure PVDF fibers.** σ, stress; ε, strain.


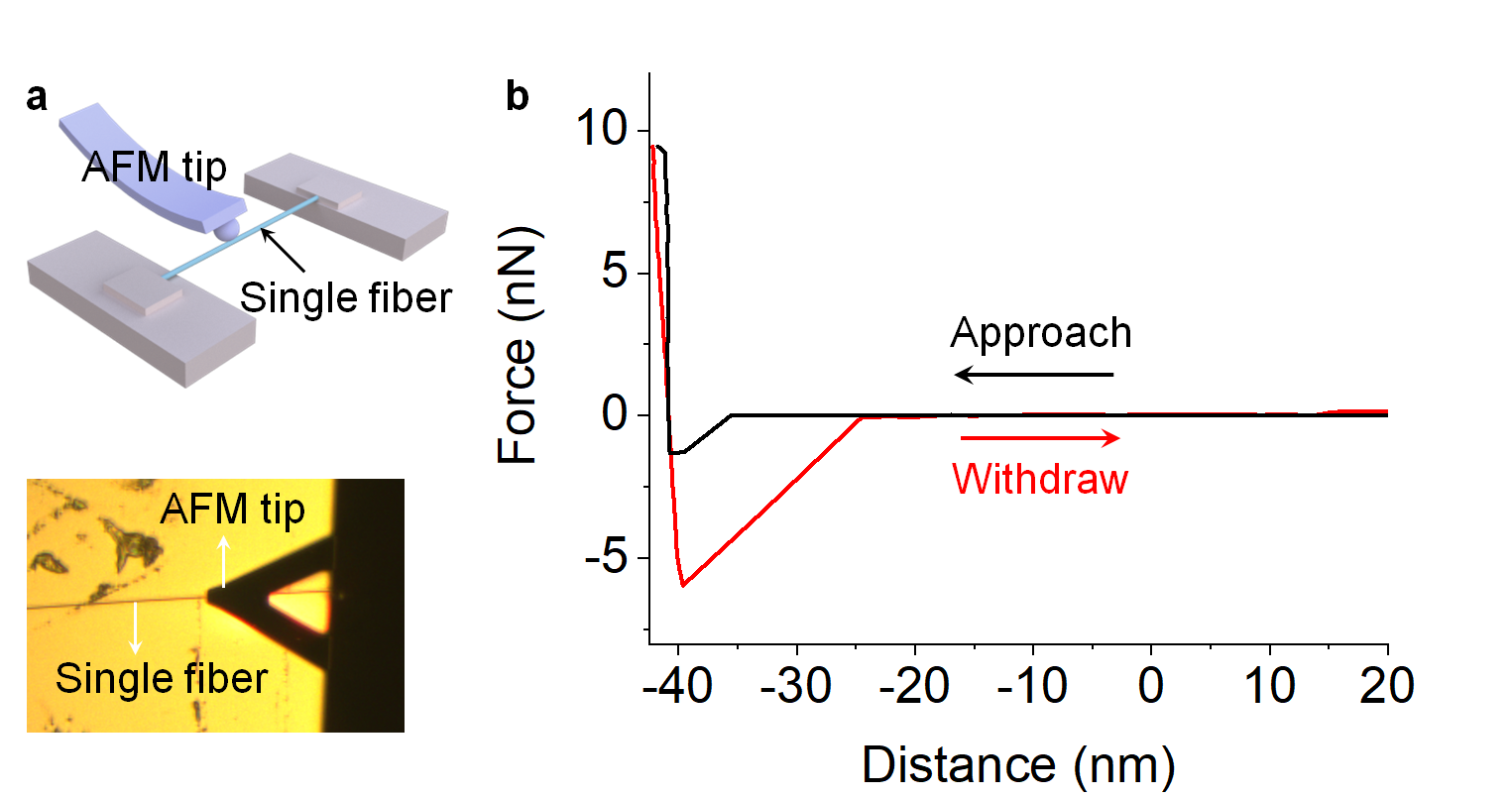


**Supplementary Fig. 10.** **Modulus testing of single fiber of ferroelectrets.** **a,** A schematic and optical image of a single fiber three-point bending test performed with an atomic force microscope (AFM). **b,** AFM nanoindentation on a single ferroelectret fiber. Young’s modulus of 2.7 GPa was calculated by fitting the indentaion curve with sphere indentaion model in Nanoscope Analysis software.


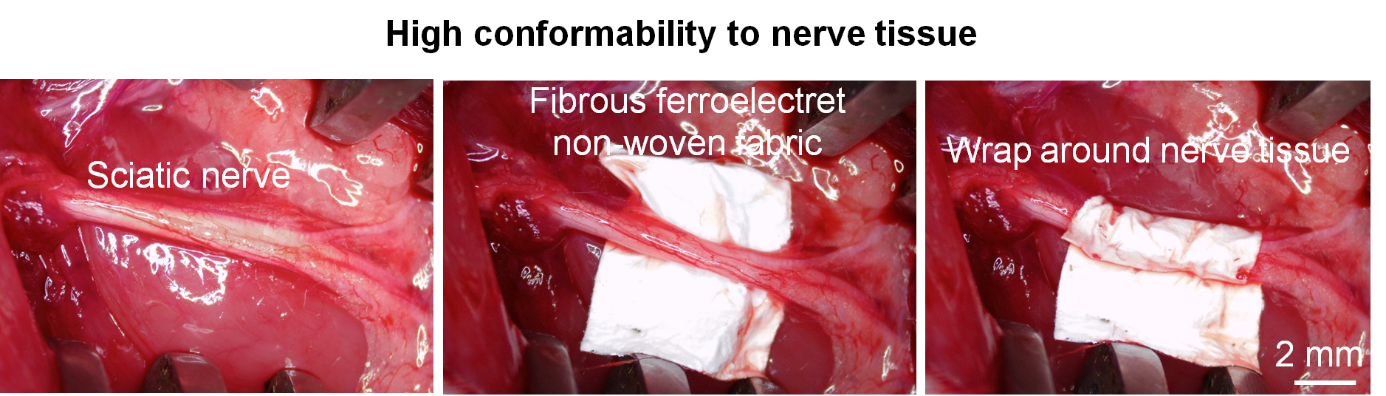


**Supplementary Fig. 11.** **High conformability to nerve tissue.** Surgical images showing that the fibrous ferroelectret non-woven fabric can be easily attached to and wrapped around the nerve tissue.


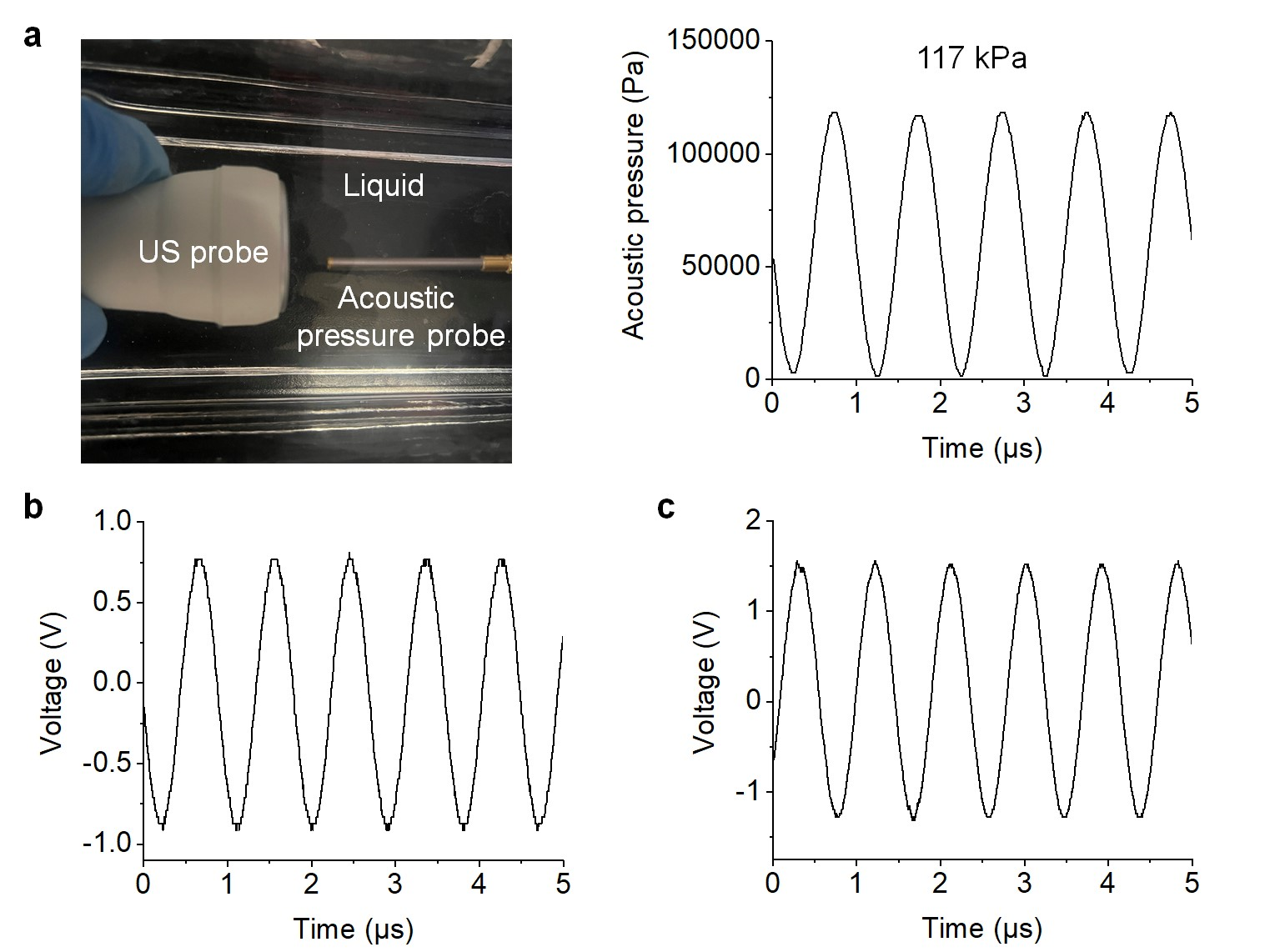


**Supplementary Fig. 12.** **Determination of ultrasound intensity.** **a,** Experimental setup (left) and acoustic pressure curve (right) measured in ethanol at a distance of 10 mm from an US probe to the acoustic pressure probe, with a US setup of 1 MHz and 0.5 W cm^−2^. Piezoelectric voltage output measured in ethanol at 10 mm from an US probe to the **(b)** air-blowing PVDF fibers and **(c)** PVDF/ZnO composite fibers, with an US setup of 1 MHz and 0.5 W cm^−2^.


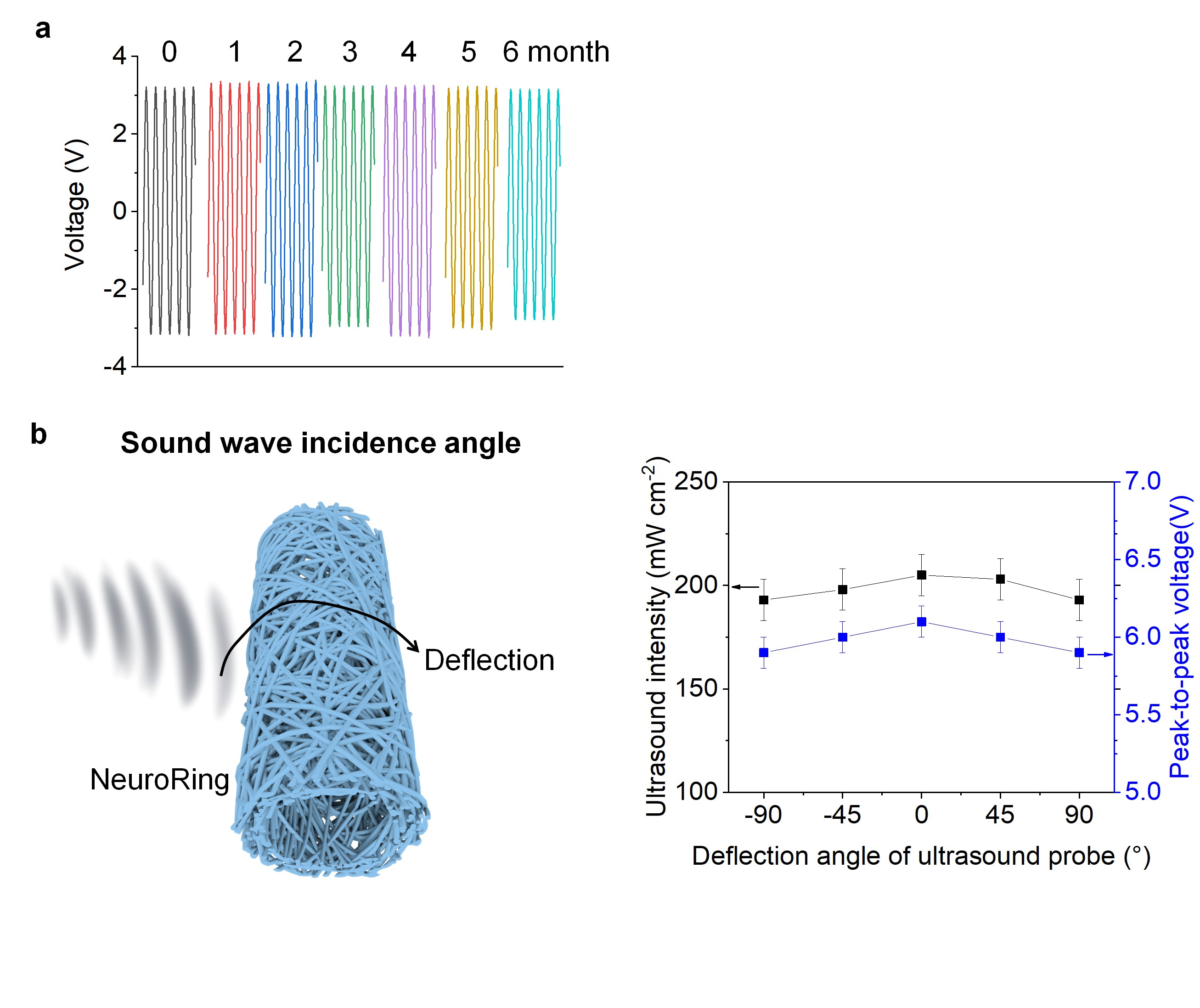


**Supplementary Fig. 13. The long-term stability and misalignment resistance of NeuroRing based on ferroelectrets. a,** Comparison of the output voltage before and after the ferroelectret fibers being immersed in a PBS solution at 37 °C for 6 months. **b,** The relationship between ultrasound intensity on ring-shaped ferroelectret film and the deflection angle of ultrasound probe. Data are expressed as mean values ± SD. All error bars indicate ± SD.


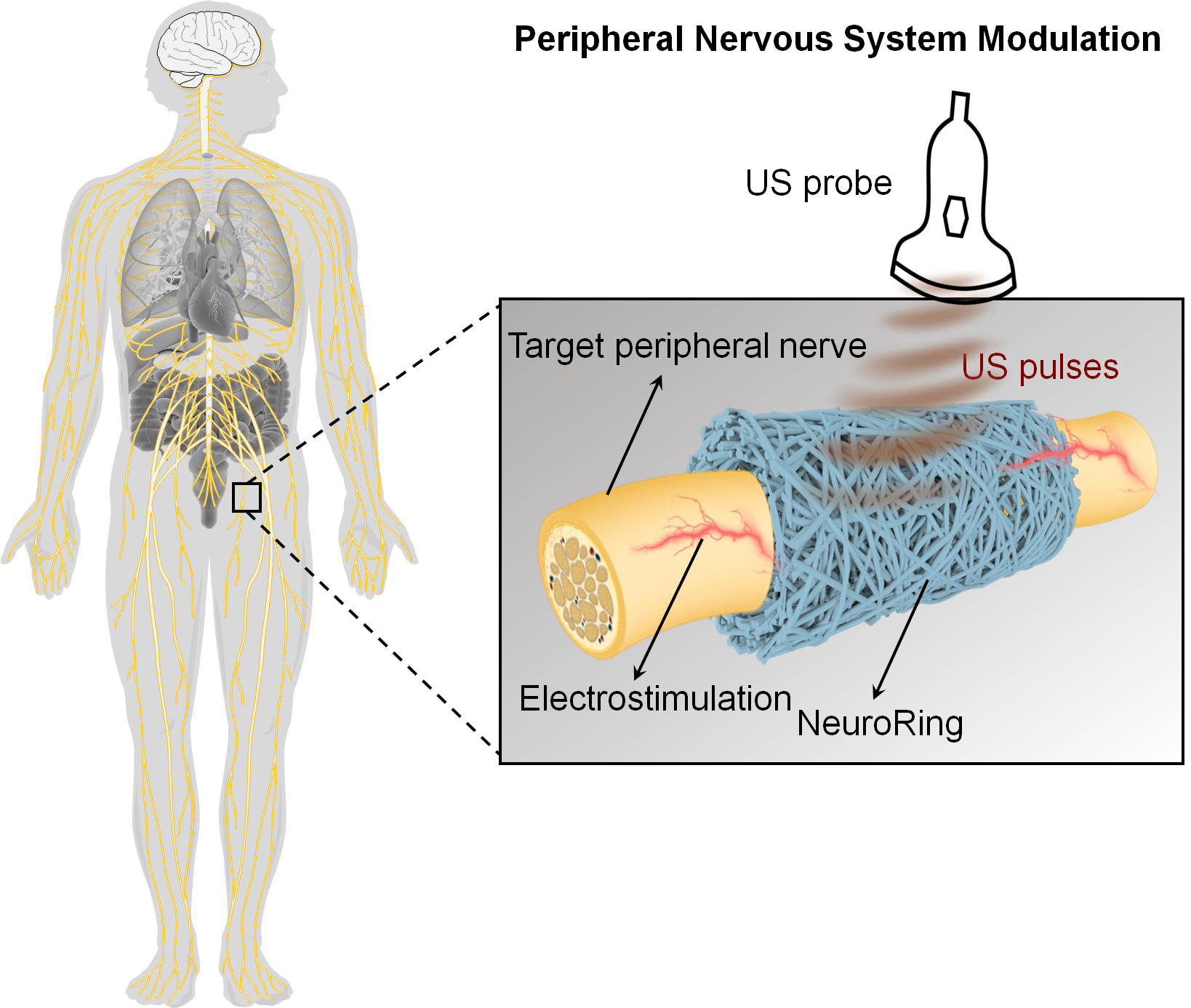


**Supplementary Fig. 14. A conceptual diagram of how the NeuroRing could be used for peripheral nerve modulation.** Peripheral nerves that branch out from the brain and spinal cord have extensive control of physiological function which can impact chronic disease. A small number of fibers per nerve makes it more amenable to targeted modulation. In our rat model, we implanted high-performance flexible ferroelectret films and wrapped target peripheral nerves to act as ultrasound receivers. Once ultrasound pulses are applied, the ultrasound receiver is activated to yield electric pulses that stimulate and modulate the nerves.


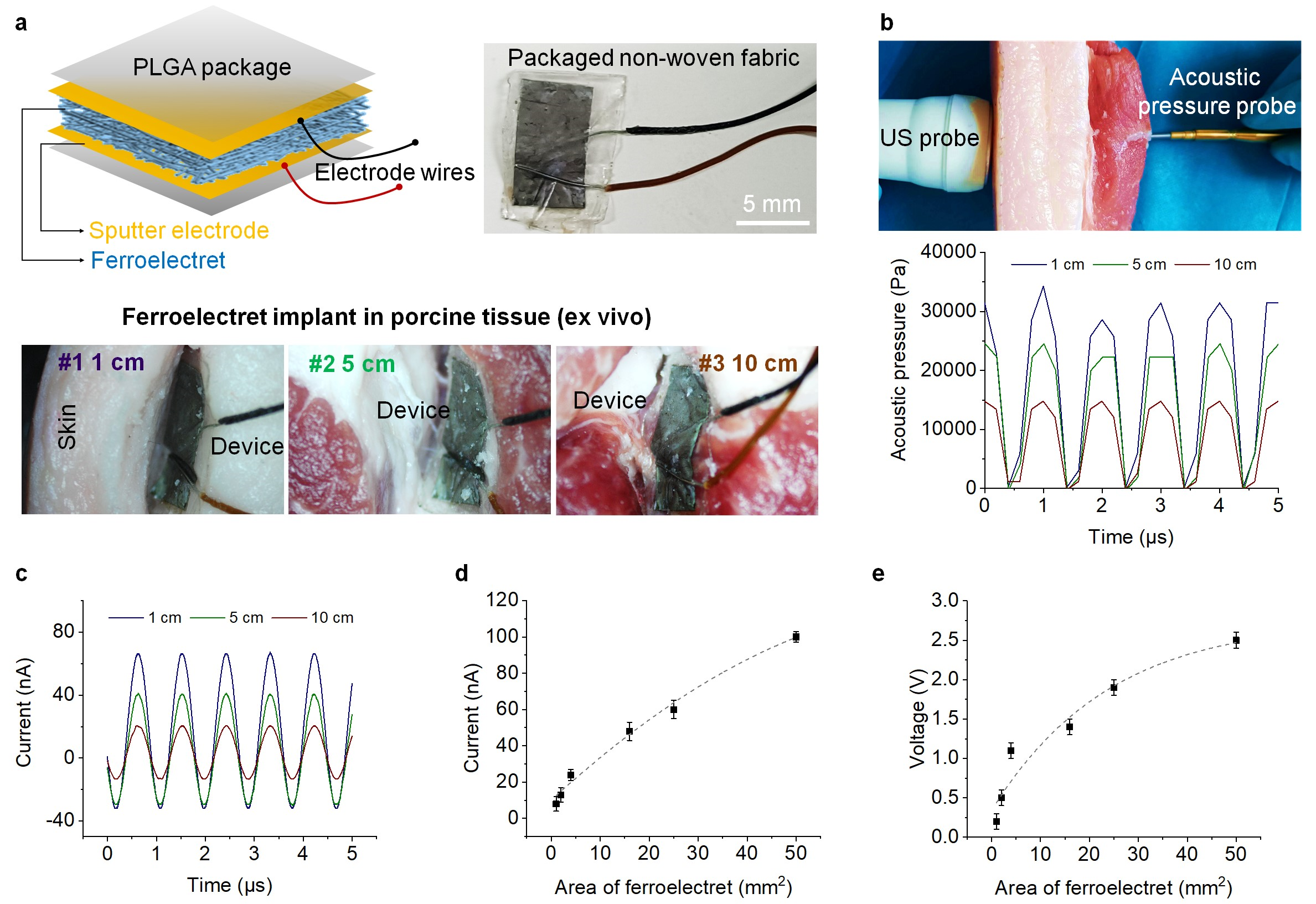


**Supplementary Fig. 15. Performance evaluation of ferroelectrets in ex vivo pork tissue.** **a,** Schematic and optical images of the package fibrous ferroelectret non-woven fabric. Bottom: Optical images of packaged ferroelectret devices implanted at 10 mm, 5 cm and 10 cm under porcine tissue. A 40-μm-thick film was fabricated by fibrous ferroelectret non-woven fabric with a size of 5 × 10 mm^2^, and gold was sputtered on the upper and lower sides as electrodes. Subsequently, the device was packaged using flexible PLGA as an encapsulation layer. **b,** Acoustic pressure applied on the ferroelectrets implanted at 1 cm, 5 cm, and 10 cm under the porcine tissue. **c,** Current generated by the ferroelectrets implanted at 1 cm, 5 cm, and 10 cm under the porcine tissue. **d,** Current outputs as a function of the ferroelectret area from 1 to 50 mm^2^. **e,** Voltage outputs as a function of the ferroelectret area from 1 to 50 mm^2^. Data are expressed as mean values ± SD. All error bars indicate ± SD.


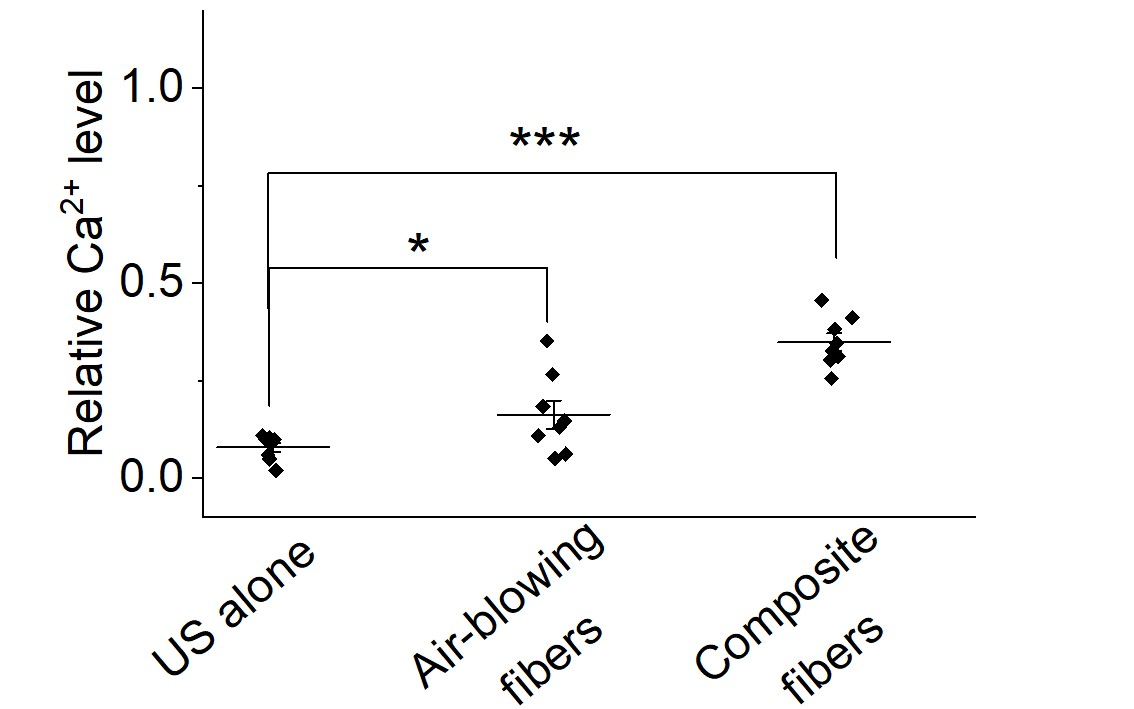


**Supplementary Fig. 16. Assessment of Ca^2+^ activation.** Relative Ca^2+^ levels of cells on air-blowing fibers and composite fibers under ultrasound stimulation. Data are expressed as mean values ± SD. All error bars indicate ± SD. n = 8 independent samples for each group. **P* < 0.05, ****P* < 0.001. US alone/Air-blowing fibers: *P* = 0.031; US alone/Composite fibers: *P* = 2.5795E-7. *P*-values are evaluated through one-sided ANOVA and post-Tukey analysis.


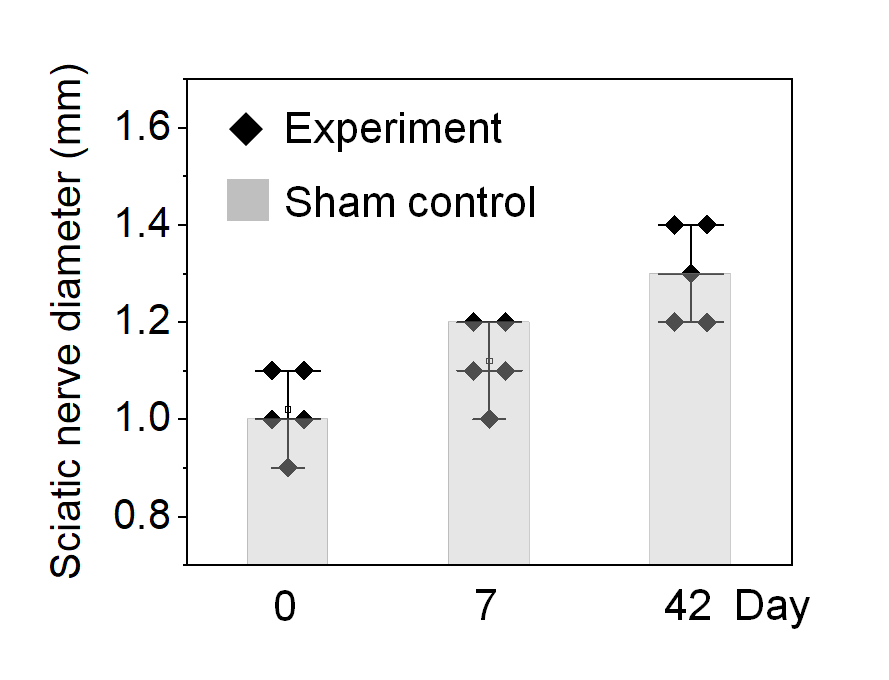


**Supplementary Fig. 17. Neurodevelopmental assessment after NeuroRing implantation.** Diameter of sciatic nerves with a NeuroRing after 42-day implantation (n = 5 biologically independent animals for each group). The diameter of the sciatic nerves with NeuroRing is almost the same as that of normal rats, indicating the rats implanted with the NeuroRing developed normally. Data are expressed as mean values ± SD. All error bars indicate ± SD.


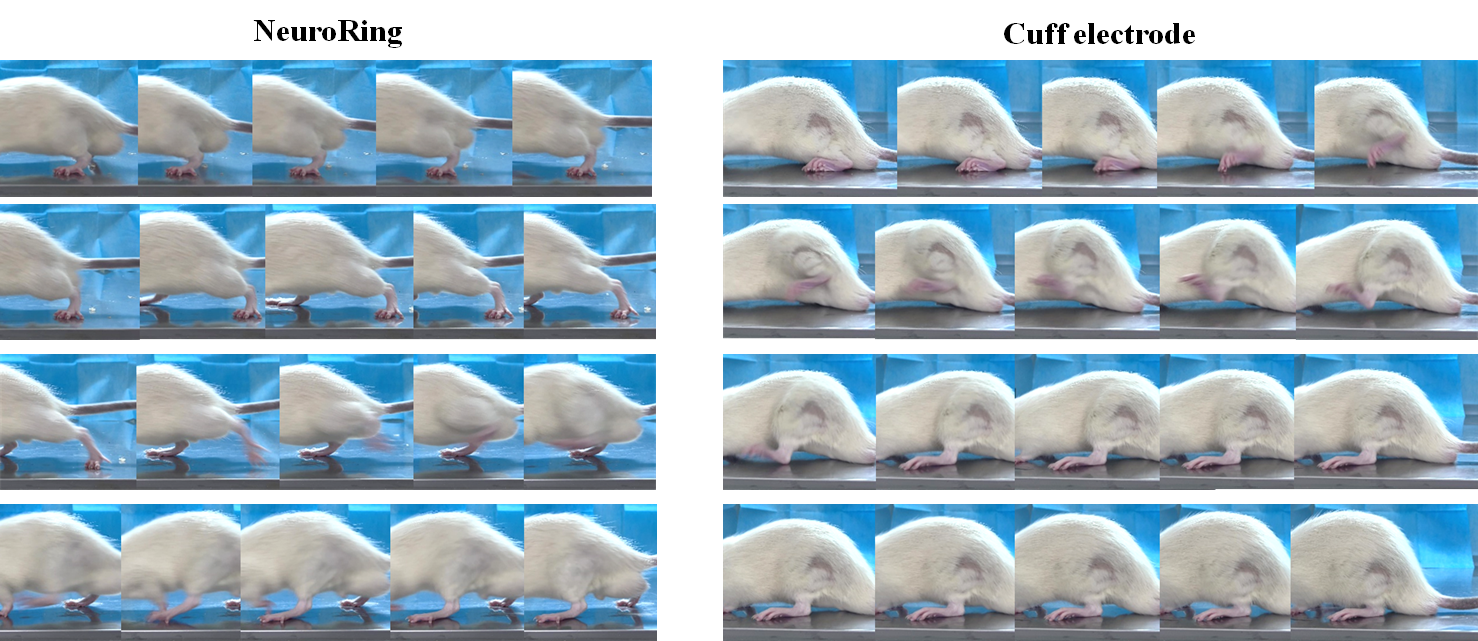


**Supplementary Fig. 18.** **Snapshot photos showing hindlimb kinematics during walking at 42-day post-implantation.** Both the stance and swing phases of the gait cycle showed observable differences between these two different implanted groups. Rats with NeuroRing demonstrated ankles in plantarflexion at the time of initial contact of the foot, while walking on a planar surface. Cuff electrode application led to an increase in the ankle peak, dorsiflexion angle, and no plantarflexion was observed during the second half of the stance phase due to nerve injury and weakening of the ankle plantar-flexor muscles. Sharp movements consisting of fast contractions and fine coordination between the ankle and joints were observed in rats with implanted NeuroRing. The passive and dragging movements in those with cuff electrodes indicated signs of sciatic-injury.


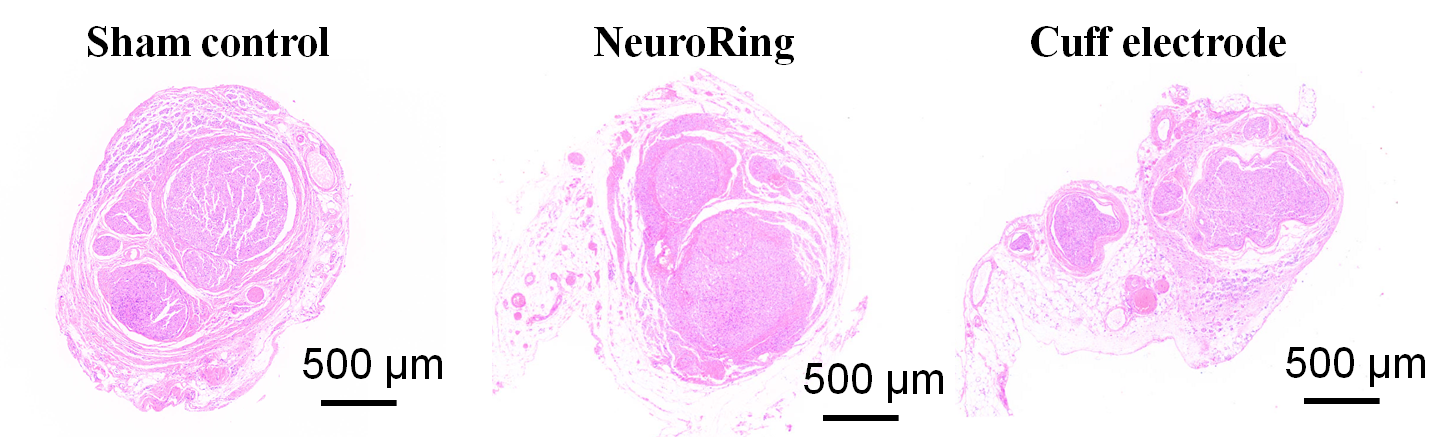


**Supplementary Fig. 19. In vivo biocompatibility assessment of NeuroRing.** H&E staining for sham control, NeuroRing and cuff electrode. Severe structural damage was observed in nerves implanted with cuff electrodes.


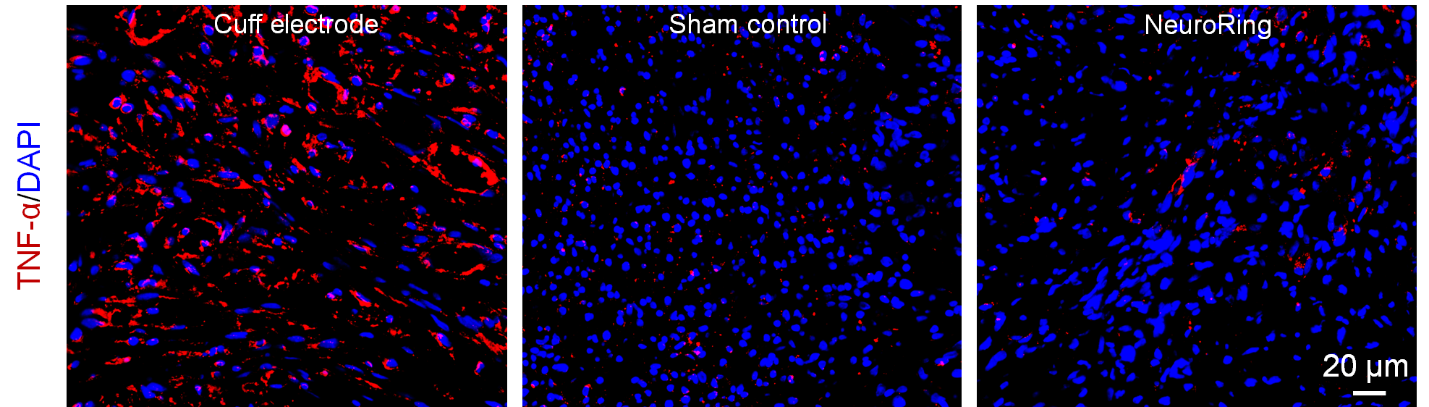


**Supplementary Fig. 20.** **In vivo assessment of inflammatory response of NeuroRing.** Cross-section slice of the sciatic nerves labelled by the inflammatory biomarker TNF-α for the cuff electrodes, sham control and NeuroRing.


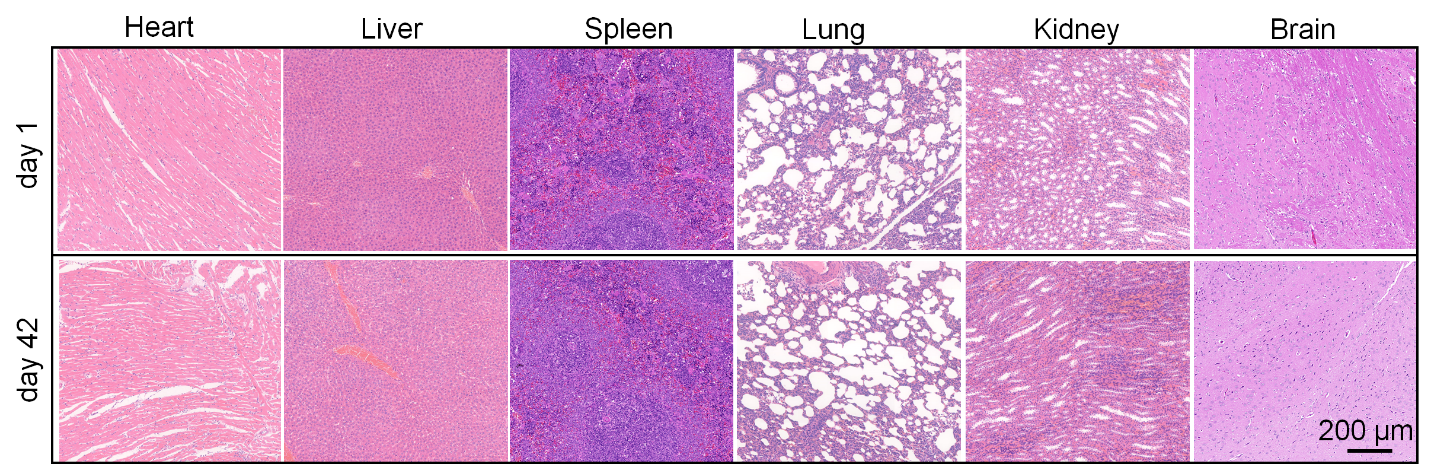


**Supplementary Fig. 21. Assessment of systemic side effects after NeuroRing implantation.** H&E staining of heart, liver, spleen, lung, kidney, and brain, at different time points (day 1, and day 42) after NeuroRing implantation.


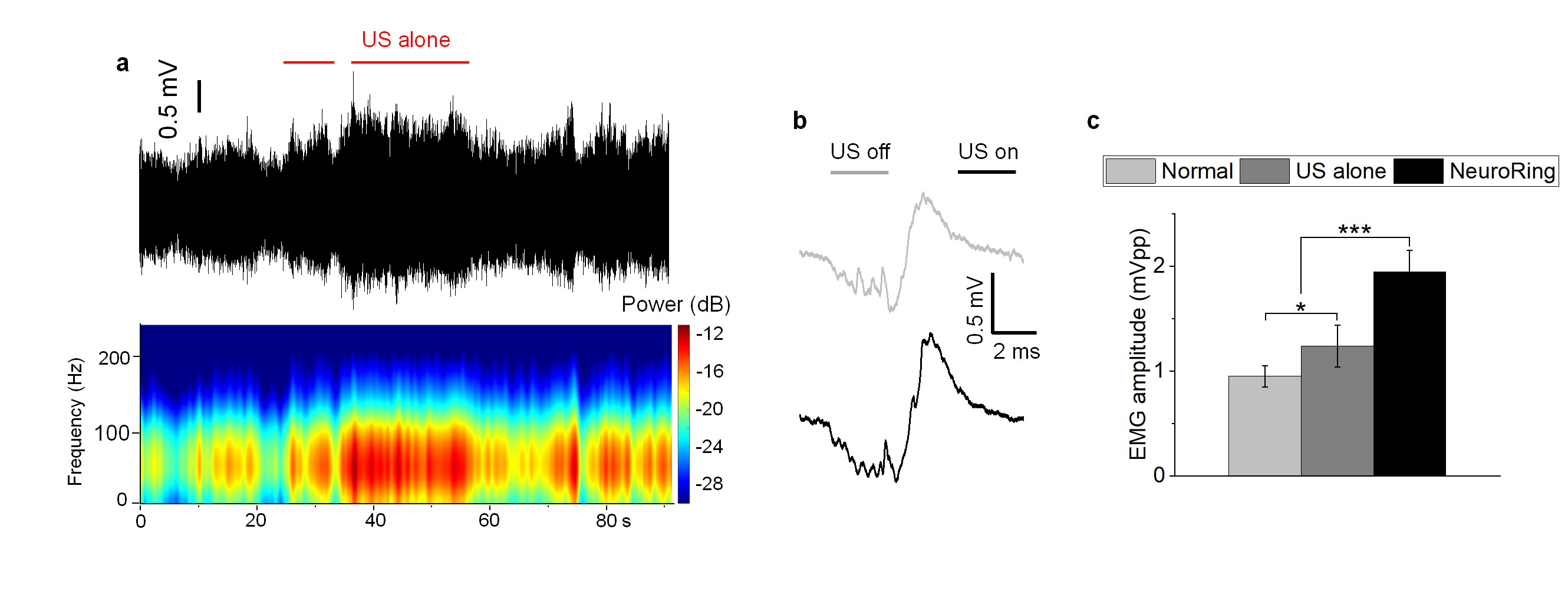


**Supplementary Fig. 22.** **Assessment of gastrocnemius activation level stimulated by US alone. a,** EMG and the corresponding power spectral analysis of US alone group. **b,** Representative EMG signals with US on and off in the US alone group. **c,** EMG voltage amplitude. Data are expressed as mean values ± SD. All error bars indicate ± SD. n = 5 for each group. *P < 0.05, ***P < 0.001. *P_Normal/US_* = 0.049294; *P_Normal/Neuro_* = 1.7E-5; *P_US/Neuro_* = 6.71E-4. *P*-values are evaluated through one-sided ANOVA and post-Tukey analysis.


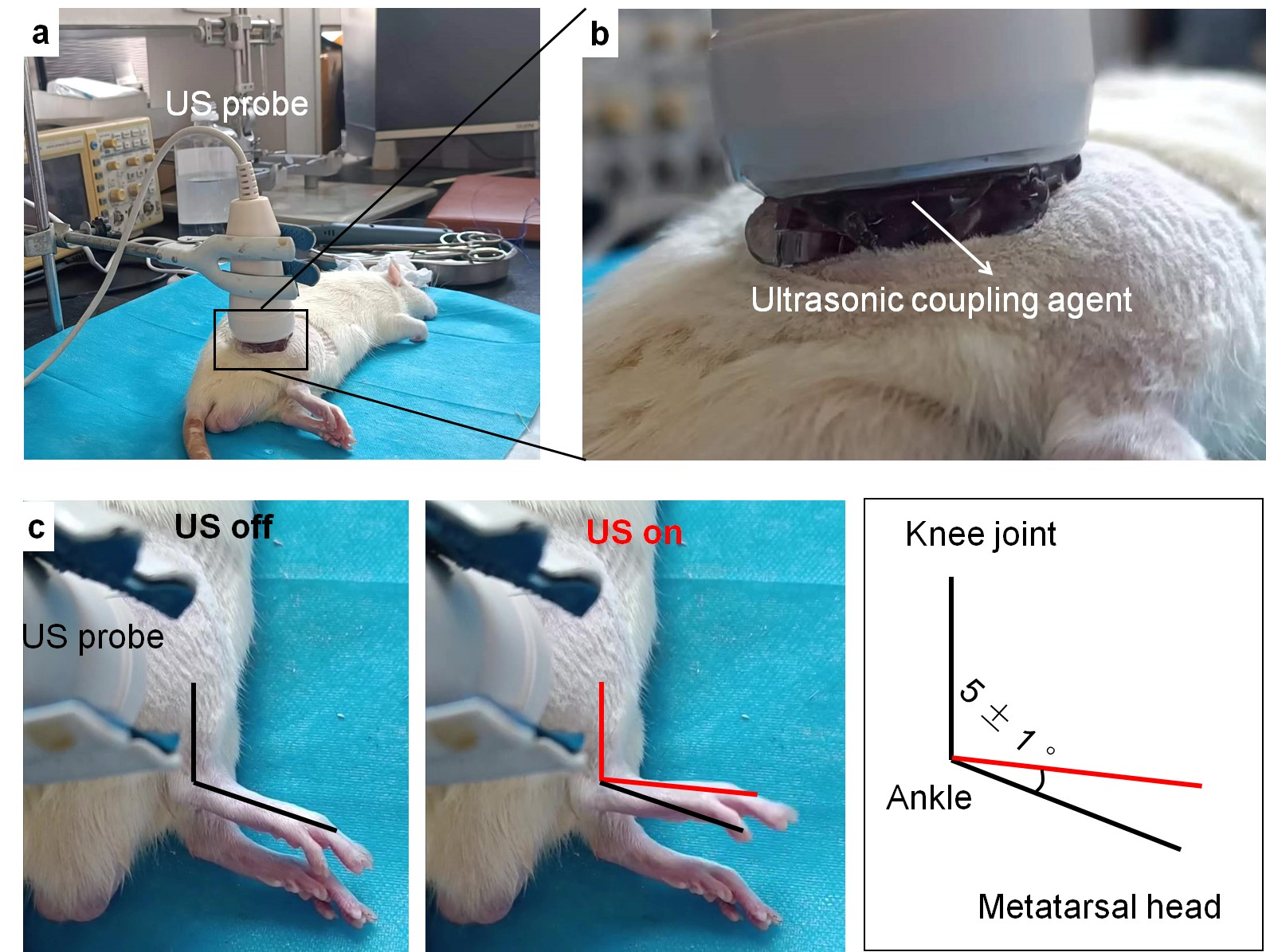


**Supplementary Fig. 23.** **Gastrocnemius activation reflected by ankle flexion.** **a,** Snapshot photo of a suspended and fixed ultrasound probe. **b,** Snapshot photo of the ultrasound coupling agent between the ultrasound probe and the rat skin. **c,** Representative images of the movement of the legs before and after stimulation. Average angular change of the ankle joint in response to stimulation.


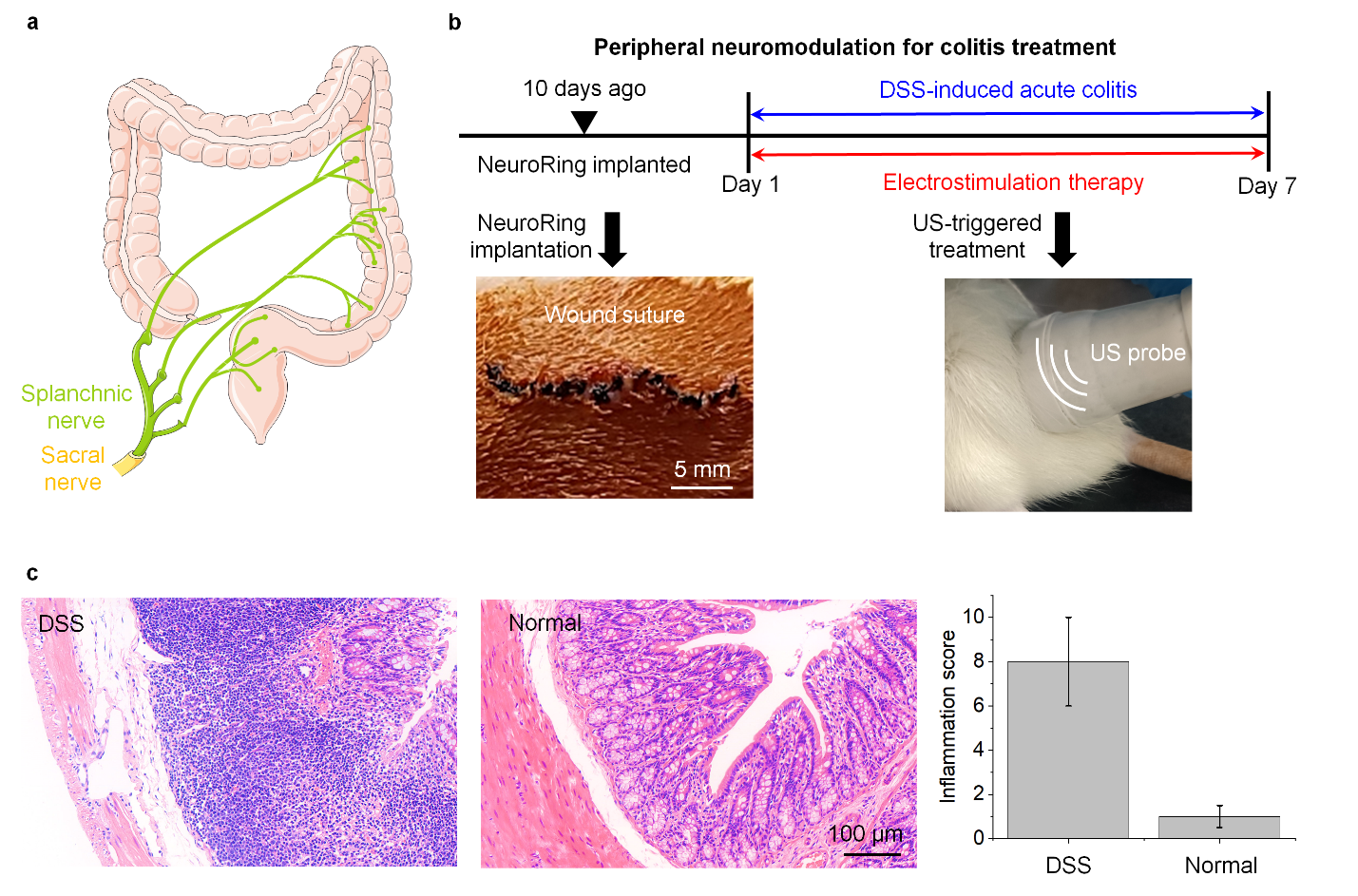


**Supplementary Fig. 24.** **Colitis modeling. a,** Schematic illustration of direct innervation of the sacral nerves to the distal colon and rectum. **b,** Schematic diagram of protocols of how the DSS-induced acute colitis rat model was established and treatment. Inset on the lower left: Surgical image of a sutured wound after NeuroRing implantation. Inset on the lower right: Optical image of the NeuroRing implanted in the sacral nerve excited by US pulses. **c,** Representative H&E staining images showing the colitis (left) with inflammation induced by DSS after 7 days of colitis development and normal rats (middle) treated with 0.9% saline. Right: Relative inflammatory expression level evaluated from left two H&E stain images. Data are expressed as mean values ± SD. All error bars indicate ± SD.


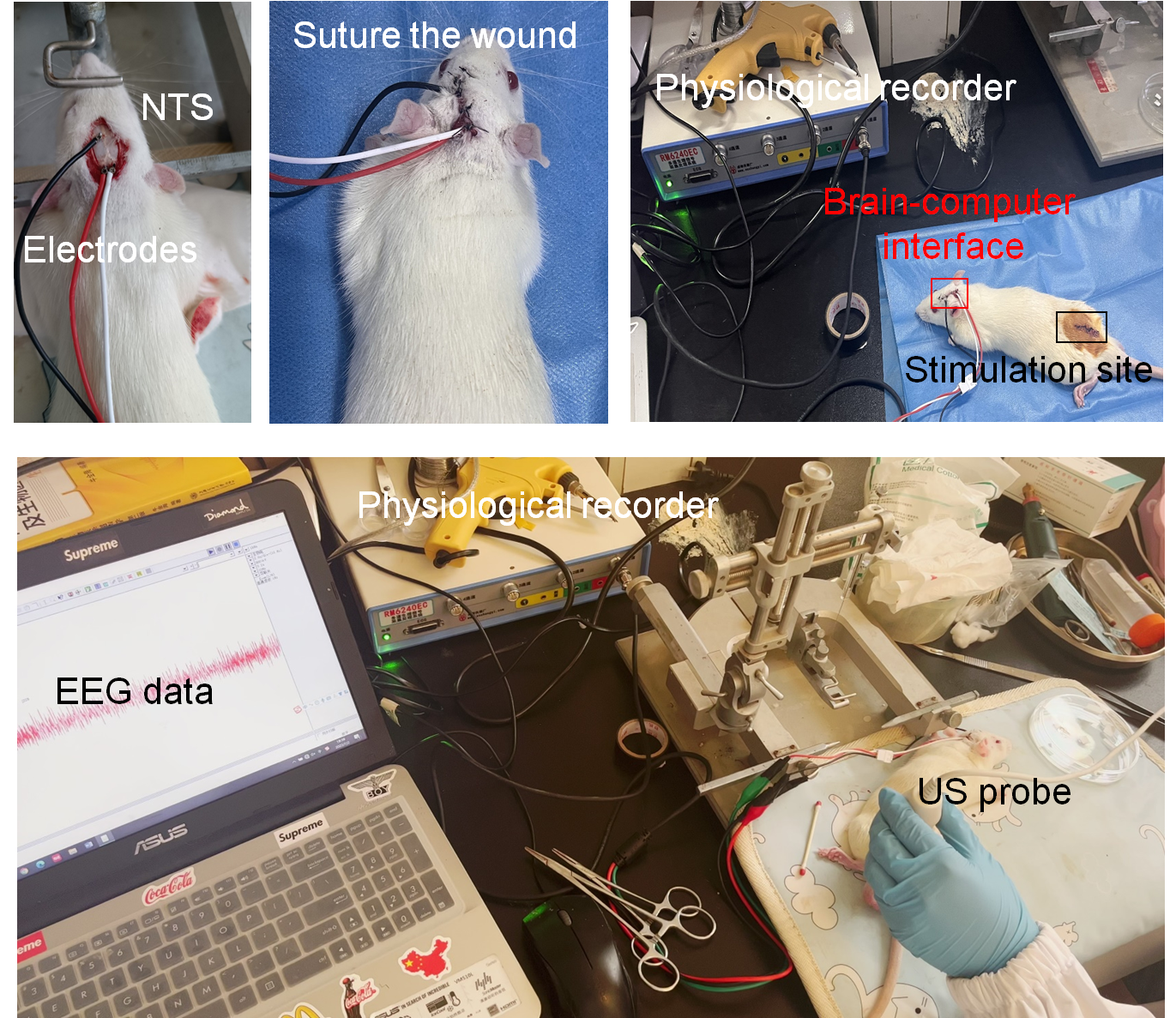


**Supplementary Fig. 25.** **Optical image of EEG signal acquisition.** When the NeuroRing is activated by ultrasound, EEG signals are collected synchronously.


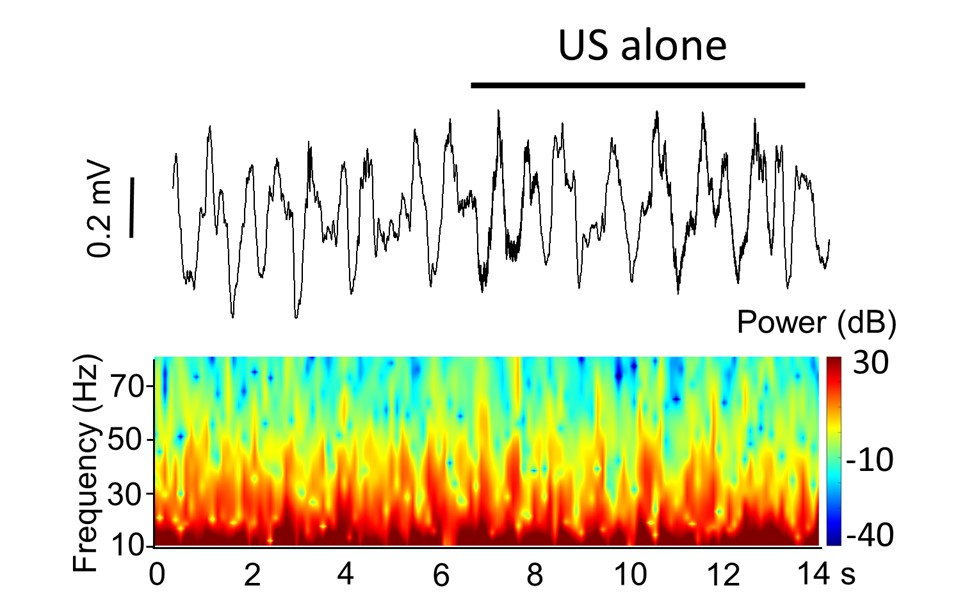


**Supplementary Fig. 26. EEG (top) recorded over parietal cortex and comparison of power spectrum (bottom) for US alone.** US alone refers to the sham operation and drinking DSS group.


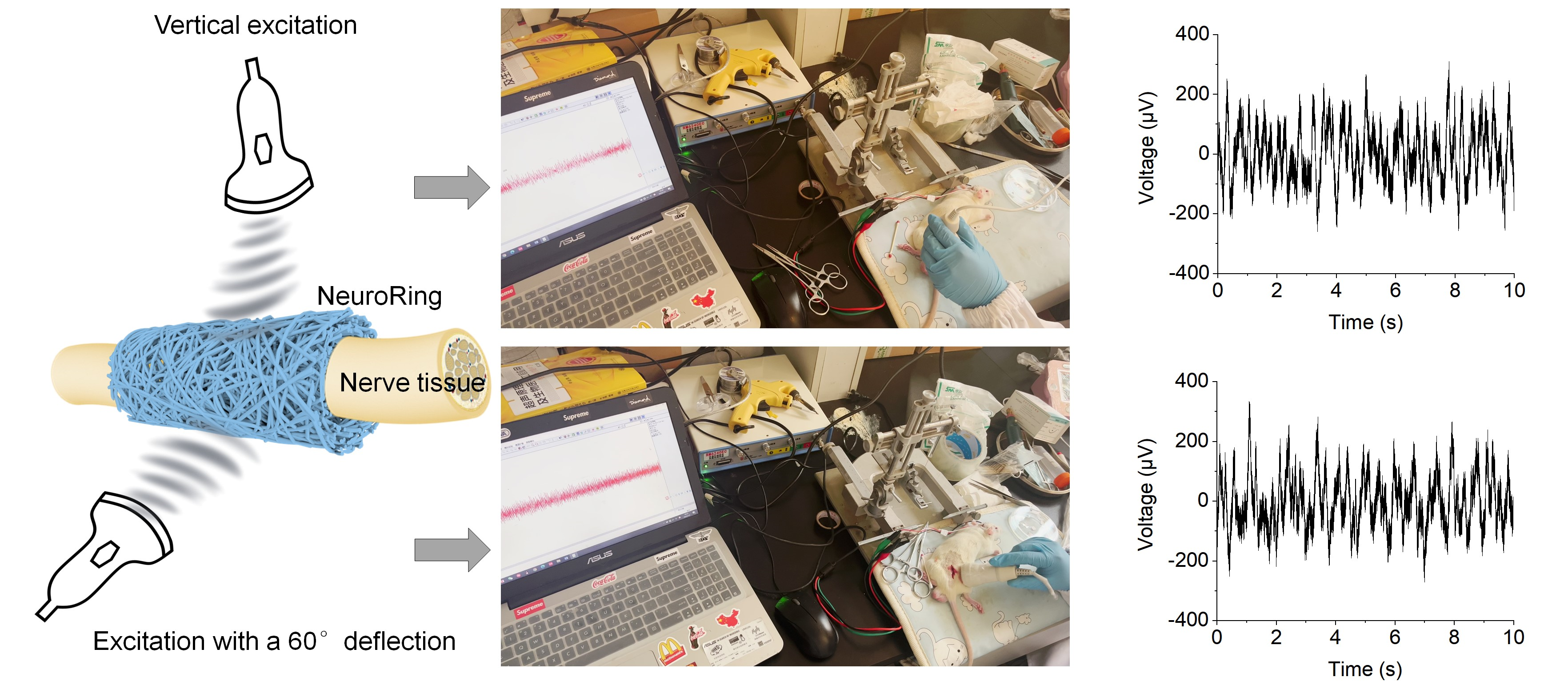


**Supplementary Fig. 27. Evaluation of NeuroRing's resistance to misalignment in vivo.** After the ultrasonic incidence angle was deflected by 60 °, the nerve was still activated, which was reflected in the unchanged EEG signal compared with the vertical excitation.


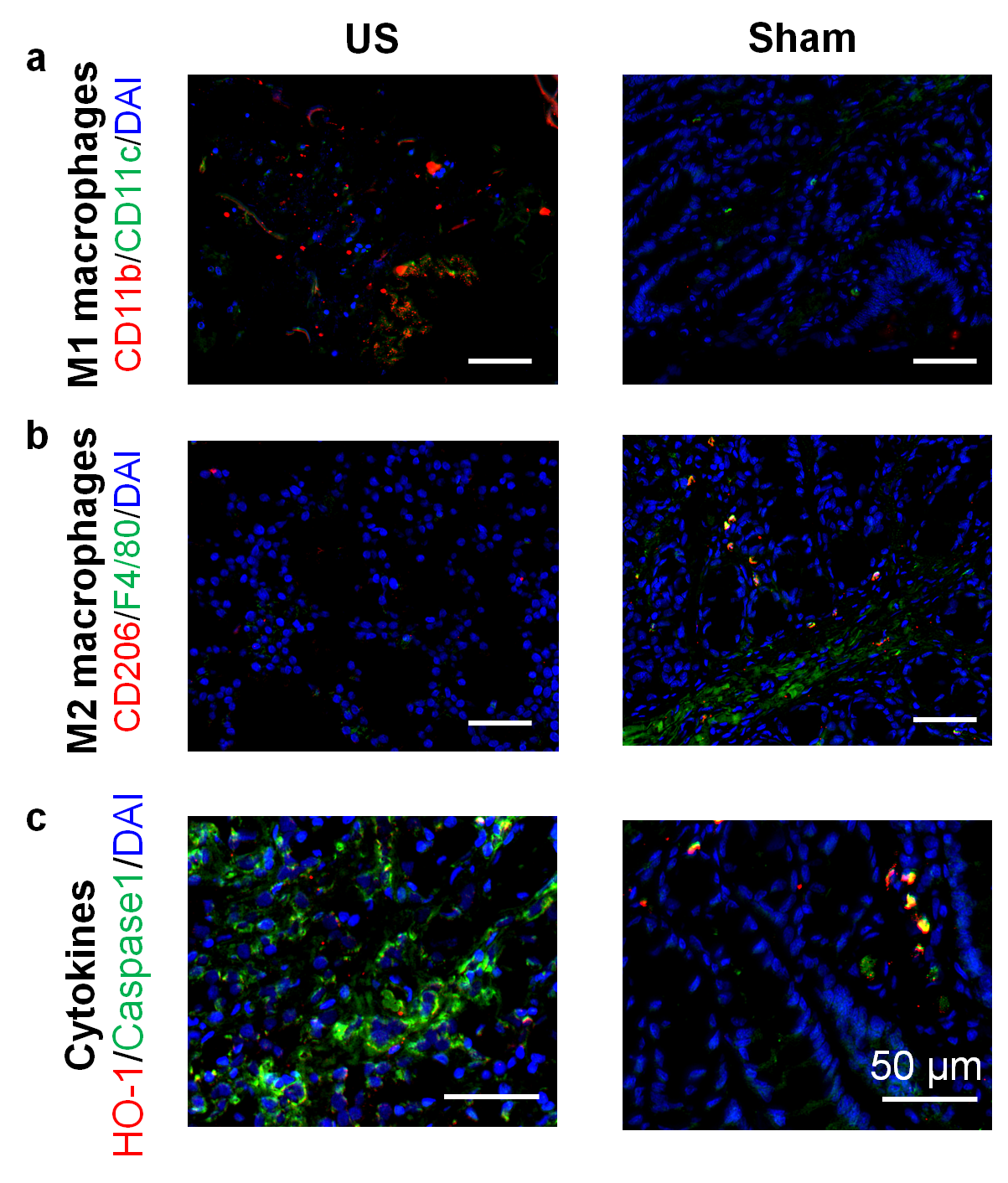


**Supplementary Fig. 28.** **Assessment of macrophages.** Immunostaining for **(a)** M1 macrophages (CD11b (red), CD11c (green)), **(b)** M2 macrophages (CD206 (red), F4/80 (green)), and **(c)** HO-1 (red) and Caspase1 (green) of US and sham control groups.


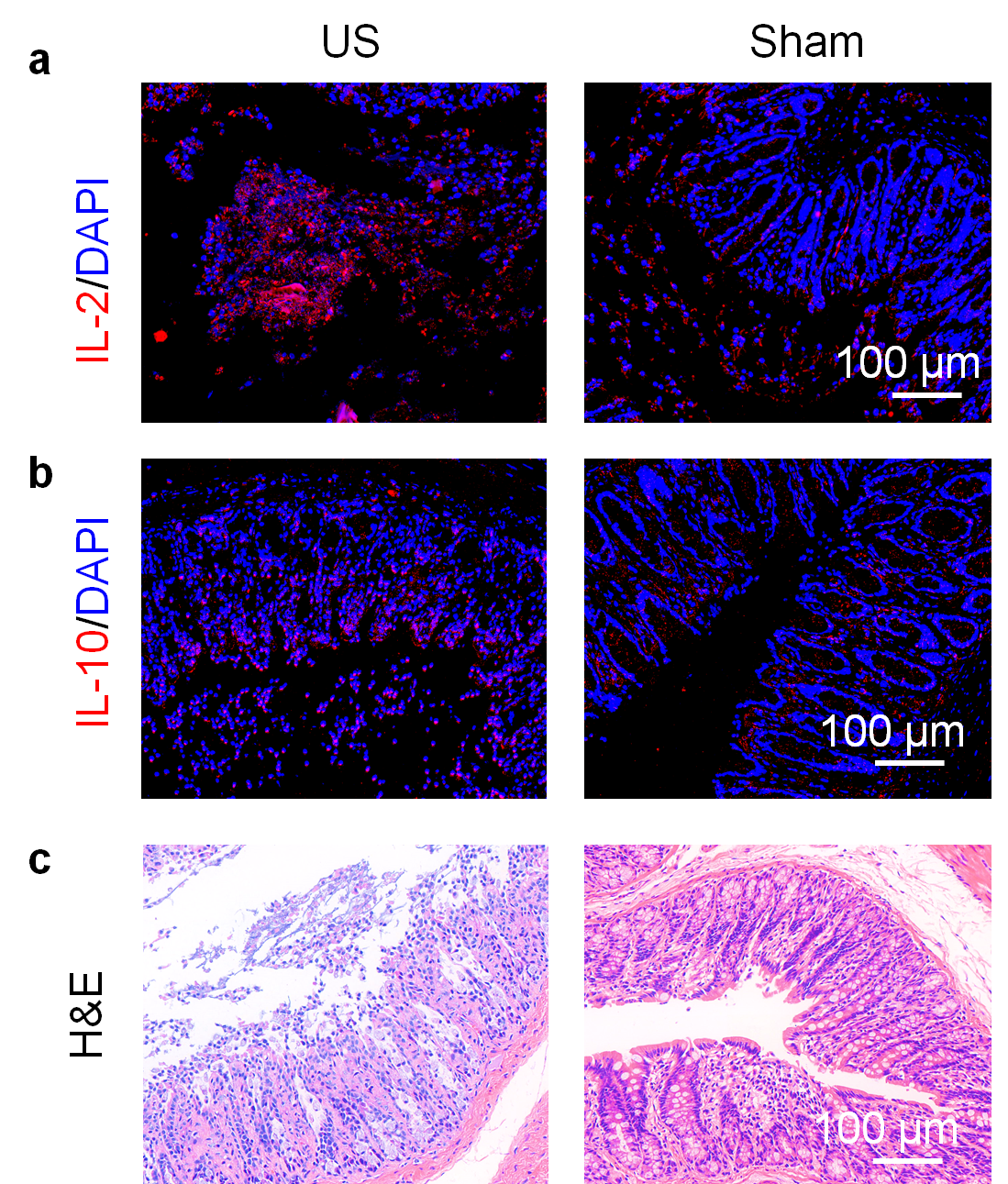


**Supplementary Fig. 29.** **Evaluation of treatment for colitis.** Immunostaining for **(a)** IL-2, and **(b)** IL-10 of US and sham control groups. **c,** H&E stained images of colon tissue in the US and sham control groups.

**Supplementary Tables**

**Supplementary Table 1. Comparison of d_33_ piezoelectric coefficient between ceramics, polymers, and state-of-the-art composites.**

| **Classification** | **Materials** | **\|d_33_\| (pC N^-1^)** | **Young's modulus (MPa)** | **Reference** |
| --- | --- | --- | --- | --- |
| **Ceramics** | Aluminum nitride (AlN) | 5 | 204,000-396,000 | 12, 13 |
|  | Barium Titanate (BaTiO_3_) | 45-191 | 80,000-200,000 | 14, 15, 16 |
|  | Lithium niobate (LiNbO_3_) | 6-16 | 140,000-355,000 | 17, 18 |
|  | Zinc oxide (ZnO) | 9.9-26.7 | 35,000-140,000 | 19, 20, 21, 22 |
|  | Potassium sodium niobate (KNN) | 138.2-180 | 100,000-180,000 | 23, 24 |
|  | KNN- LiSbO_3_ | 283 | 64,940 | 25, 26 |
|  | Lead Zirconate Titanate (PZT) | 90-870 | 70,000-139,000 | 27 |
|  | Aerosol-deposited PZT | 406 | 60,000 | 28 |
|  | Micro-fabricated PZT | / | 3,400 | 29 |
|  | Bismuth sodium titanate (BNT) | 12.5-150 | 100,000 | 30, 31 |
|  | Cadmium sulfide (CdS) | 2.56, 10.65 | 70,000 | 32, 33 |
| **Polymers** | Glycine | 5.3 | 2000-9000 | 34 |
|  | Cellulose | 19.3 | 15,000-20,000 | 35, 36, 37 |
|  | Parylene-C | 1-2 | 4500 | 38, 39 |
|  | β-CN | 16.5 | 2500 | 40 |
|  | Diphenylalanine (DPA) | 17.9 | 19,000-27,000 | 41 |
|  | Poly(Lactic Acid) (PLLA) | 7-12 | 1.06-20,000 | 42, 43, 44, 45 |
|  | PVDF-TrFE | 25-40 | 1,500 | 46, 47 |
|  | Nylon-11 | 7.2 | / | 48 |
|  | Polyimide | 2.7 | / | 49 |
|  | PVDF film | 13-58.5 | 400-1800 | 50, 51, 52, 53, 54, 55, 56, 57 |
|  | Porous PVDF | / | 14.6-121 | 58 |
|  | PVDF fibers | 25-28 | 1.64-91.8 | 59, 60 |
| **Composites** | KNN-PVDF | 12 | 470-1480 | 61, 62 |
|  | PVDF/BaTiO_3_ | 48 | 2170-3030 | 63, 64 |
|  | PVDF/ZnO | 31.4 | 2170 | 65 |
|  | PVDF/PZT | 35 | 1500-2000 | 66 |
|  | Polypropylene/Silicates | / | 1500 | 67 |
|  | PVDF/PDMS | / | 0.8-30.16 | 68 |
|  | P(VDF-TrFE)/BaTiO_3_ | 46 | 784 | 69, 70 |
|  | **PVDF/ZnO fibers** | **56 ± 2** | **7.6** | **This work** |

**Supplementary Table 2. Histological scoring system.**

| Score | Inflammation | Mucosal damage | Crypt damage | Range of lesions (%) |
| --- | --- | --- | --- | --- |
| 0 | None | None | None | 0 |
| 1 | Mile | Mucous layer | 1/3 | 1-25 |
| 2 | Moderate | Submucosa | 2/3 | 26-50 |
| 3 | Severe | Muscularis and serosa | 100% | 51-75 |
| 4 | - | - | 100% + epithelium loss | 76-100 |

**Supplementary Table 3. DAI scoring system.**

| DAI score | Weight loss (%) | Stool consistency | Occult/gross bleeding |
| --- | --- | --- | --- |
| 0 | None | Normal | Normal |
| 1 | 1-5 | Loose stools | Hemoccult positive |
| 2 | 5-10 |  |  |
| 3 | 10-20 |  |  |
| 4 | >20 | Diarrhea | Gross bleeding |

**Supplementary References**

1. Liu D*, et al.* Cavitation in strained polyethylene/aluminium oxide nanocomposites. *European Polymer Journal* **87**, 255-265 (2017).

2. Perchacz M, Rozanski A, Kargarzadeh H, Galeski A. Cavitation in high density polyethylene/Al2O3 nanocomposites. *Composites Science and Technology* **199**, (2020).

3. Liu D, Pourrahimi AM, Olsson RT, Hedenqvist MS, Gedde UW. Influence of nanoparticle surface treatment on particle dispersion and interfacial adhesion in low-density polyethylene/aluminium oxide nanocomposites. *European Polymer Journal* **66**, 67-77 (2015).

4. Huang Y*, et al.* Enhanced piezoelectricity from highly polarizable oriented amorphous fractions in biaxially oriented poly(vinylidene fluoride) with pure beta crystals. *Nat Commun* **12**, 675 (2021).

5. Zhu Z*, et al.* Electrostriction-enhanced giant piezoelectricity via relaxor-like secondary crystals in extended-chain ferroelectric polymers. *Matter* **4**, 3696-3709 (2021).

6. Li T*, et al.* High-Performance Poly(vinylidene difluoride)/Dopamine Core/Shell Piezoelectric Nanofiber and Its Application for Biomedical Sensors. *Adv Mater* **33**, e2006093 (2021).

7. Zhang Z, Yao C, Yu Y, Hong Z, Zhi M, Wang X. Mesoporous Piezoelectric Polymer Composite Films with Tunable Mechanical Modulus for Harvesting Energy from Liquid Pressure Fluctuation. *Advanced Functional Materials* **26**, 6760-6765 (2016).

8. Zhu Z, Rui G, Li R, He H, Zhu L. Effect of Dipole Mobility in Secondary Crystals on Piezoelectricity of a Poly(vinylidene fluoride-co-trifluoroethylene) 52/48 mol % Random Copolymer with an Extended-Chain Crystal Structure. *Macromolecules* **54**, 9879-9887 (2021).

9. Mohebbi A, Mighri F, Ajji A, Rodrigue D. Cellular Polymer Ferroelectret: A Review on Their Development and Their Piezoelectric Properties. *Advances in Polymer Technology* **37**, 468-483 (2016).

10. McFee JH, Bergman JG, Crane GR. Pyroelectric and Nonlinear Optical Properties of Poled Polyvinylidene Fluoride Films. *IEEE Transactions on Sonics and Ultrasonics* **19**, 305-314 (1972).

11. Kawai H. The Piezoelectricity of Poly (vinylidene Fluoride). *Japanese Journal of Applied Physics* **8**, (1969).

12. Österlund E, Kinnunen J, Rontu V, Torkkeli A, Paulasto-Kröckel M. Mechanical properties and reliability of aluminum nitride thin films. *Journal of Alloys and Compounds* **772**, 306-313 (2019).

13. Stoppel F, Schröder C, Senger F, Wagner B, Benecke W. AlN-based piezoelectric micropower generator for low ambient vibration energy harvesting. *Procedia Engineering* **25**, 721-724 (2011).

14. Wang F, Mai Y-W, Wang D, Ding R, Shi W. High quality barium titanate nanofibers for flexible piezoelectric device applications. *Sensors and Actuators A: Physical* **233**, 195-201 (2015).

15. Cordero F. Quantitative evaluation of the piezoelectric response of unpoled ferroelectric ceramics from elastic and dielectric measurements: Tetragonal BaTiO3. *Journal of Applied Physics* **123**, (2018).

16. Maruyama K, Kawakami Y, Narita F. Young’s modulus and ferroelectric property of BaTiO3 films formed by aerosol deposition in consideration of residual stress and film thickness. *Japanese Journal of Applied Physics* **61**, (2022).

17. Hossain MM. First-principles study on the structural, elastic, electronic and optical properties of LiNbO(3). *Heliyon* **5**, e01436 (2019).

18. Smith RT, Welsh FS. Temperature Dependence of the Elastic, Piezoelectric, and Dielectric Constants of Lithium Tantalate and Lithium Niobate. *Journal of Applied Physics* **42**, 2219-2230 (1971).

19. Christman JA, Woolcott RR, Kingon AI, Nemanich RJ. Piezoelectric measurements with atomic force microscopy. *Applied Physics Letters* **73**, 3851-3853 (1998).

20. Zhao M-H, Wang Z-L, Mao SX. Piezoelectric Characterization of Individual Zinc Oxide Nanobelt Probed by Piezoresponse Force Microscope. *Nano Letters* **4**, 587-590 (2004).

21. Manoharan MP, Desai AV, Neely G, Haque MA. Synthesis and Elastic Characterization of Zinc Oxide Nanowires. *Journal of Nanomaterials* **2008**, 1-7 (2008).

22. Kim H*, et al.* Young's modulus of ZnO microwires determined by various mechanical measurement methods. *Current Applied Physics* **14**, 166-170 (2014).

23. Shibata K, Watanabe K, Kuroda T, Osada T. KNN lead-free piezoelectric films grown by sputtering. *Applied Physics Letters* **121**, (2022).

24. Hao J, Ye C, Shen B, Zhai J. Enhanced piezoelectric properties of 〈001〉 textured lead-free (KxNa1 − x)0.946Li0.054NbO3 ceramics with large strain. *physica status solidi (a)* **209**, 1343-1349 (2012).

25. Vaish R. Piezoelectric and Pyroelectric Materials Selection. *International Journal of Applied Ceramic Technology* **10**, 682-689 (2013).

26. Habib M, Lantgios I, Hornbostel K. A review of ceramic, polymer and composite piezoelectric materials. *Journal of Physics D: Applied Physics* **55**, (2022).

27. Luo Y*, et al.* Nanoshell tubes of ferroelectric lead zirconate titanate and barium titanate. *Applied Physics Letters* **83**, 440-442 (2003).

28. Hwang G-T*, et al.* Self-Powered Wireless Sensor Node Enabled by an Aerosol-Deposited PZT Flexible Energy Harvester. *Advanced Energy Materials* **6**, (2016).

29. Cho H, Park J, Park JY. Micro-fabricated flexible PZT cantilever using d33 mode for energy harvesting. *Micro and Nano Systems Letters* **5**, (2017).

30. Dittmer R, Jo W, Webber KG, Jones JL, Rödel J. Local structure change evidenced by temperature-dependent elastic measurements: Case study on Bi1/2Na1/2TiO3-based lead-free relaxor piezoceramics. *Journal of Applied Physics* **115**, (2014).

31. Zou H, Sui Y, Zhu X, Liu B, Xue J, Zhang J. Texture development and enhanced electromechanical properties in <00l>-textured BNT-based materials. *Materials Letters* **184**, 139-142 (2016).

32. Deligoz E, Colakoglu K, Ciftci Y. Elastic, electronic, and lattice dynamical properties of CdS, CdSe, and CdTe. *Physica B: Condensed Matter* **373**, 124-130 (2006).

33. Schofield D, Brown RF. An Investigation of Some Barium Titanate Compositions for Transducer Applications. *Canadian Journal of Physics* **35**, 594-607 (1957).

34. Yang F*, et al.* Wafer-scale heterostructured piezoelectric bio-organic thin films. *Science* **373**, 337-342 (2021).

35. Wang J*, et al.* Piezoelectric Nanocellulose Thin Film with Large-Scale Vertical Crystal Alignment. *ACS Appl Mater Interfaces* **12**, 26399-26404 (2020).

36. Quesada Cabrera R, Meersman F, McMillan PF, Dmitriev V. Nanomechanical and structural properties of native cellulose under compressive stress. *Biomacromolecules* **12**, 2178-2183 (2011).

37. Tanpichai S*, et al.* Effective Young's modulus of bacterial and microfibrillated cellulose fibrils in fibrous networks. *Biomacromolecules* **13**, 1340-1349 (2012).

38. Ramadan KS, Sameoto D, Evoy S. A review of piezoelectric polymers as functional materials for electromechanical transducers. *Smart Materials and Structures* **23**, (2014).

39. Sim W, Kim B, Choi B, Park JO. Theoretical and experimental studies on the parylene diaphragms for microdevices. *Microsystem Technologies* **11**, 11-15 (2005).

40. Park C, Ounaies Z, Wise KE, Harrison JS. In situ poling and imidization of amorphous piezoelectric polyimides. *Polymer* **45**, 5417-5425 (2004).

41. Nguyen V, Zhu R, Jenkins K, Yang R. Self-assembly of diphenylalanine peptide with controlled polarization for power generation. *Nat Commun* **7**, 13566 (2016).

42. Jariyavidyanont K*, et al.* Young's modulus of the different crystalline phases of poly (l-lactic acid). *J Mech Behav Biomed Mater* **137**, 105546 (2023).

43. Fu W*, et al.* Electrospun gelatin/PCL and collagen/PLCL scaffolds for vascular tissue engineering. *Int J Nanomedicine* **9**, 2335-2344 (2014).

44. Promnil S, Numpaisal P-o, Ruksakulpiwat Y. Effect of molecular weight on mechanical properties of electrospun poly (lactic acid) fibers for meniscus tissue engineering scaffold. *Materials Today: Proceedings* **47**, 3496-3499 (2021).

45. Farahani A, Zarei-Hanzaki A, Abedi HR, Tayebi L, Mostafavi E. Polylactic Acid Piezo-Biopolymers: Chemistry, Structural Evolution, Fabrication Methods, and Tissue Engineering Applications. *J Funct Biomater* **12**, (2021).

46. Kepler RG, Anderson RA. Ferroelectric polymers. *Advances in Physics* **41**, 1-57 (1992).

47. Fang F, Shan SC, Yang W. A multipeak phenomenon of magnetoelectric coupling in Terfenol-D/P(VDF-TrFE)/Terfenol-D laminates. *Journal of Applied Physics* **108**, (2010).

48. Wu J, Fu Y, Hu GH, Wang S, Xiong C. Effect of Stretching on Crystalline Structure, Ferroelectric and Piezoelectric Properties of Solution-Cast Nylon-11 Films. *Polymers (Basel)* **13**, (2021).

49. Simpson J, Ounaies Z, Fay C. Polarization and Piezoelectric Properties of a Nitrile Substituted Polyimide. *MRS Proceedings* **459**, (2011).

50. Kanik M, Aktas O, Sen HS, Durgun E, Bayindir M. Spontaneous high piezoelectricity in poly(vinylidene fluoride) nanoribbons produced by iterative thermal size reduction technique. *ACS Nano* **8**, 9311-9323 (2014).

51. Mokhtari F*, et al.* Wearable Electronic Textiles from Nanostructured Piezoelectric Fibers. *Advanced Materials Technologies* **5**, (2020).

52. Soin N*, et al.* Exclusive self-aligned beta-phase PVDF films with abnormal piezoelectric coefficient prepared via phase inversion. *Chem Commun (Camb)* **51**, 8257-8260 (2015).

53. Jean-Mistral C, Basrour S, Chaillout JJ. Comparison of electroactive polymers for energy scavenging applications. *Smart Materials and Structures* **19**, (2010).

54. Sharma T, Je S-S, Gill B, Zhang JXJ. Patterning piezoelectric thin film PVDF–TrFE based pressure sensor for catheter application. *Sensors and Actuators A: Physical* **177**, 87-92 (2012).

55. Chunyan L, Pei-Ming W, Soohyun L, Gorton A, Schulz MJ, Ahn CH. Flexible Dome and Bump Shape Piezoelectric Tactile Sensors Using PVDF-TrFE Copolymer. *Journal of Microelectromechanical Systems* **17**, 334-341 (2008).

56. Guo S, Duan X, Xie M, Aw KC, Xue Q. Composites, Fabrication and Application of Polyvinylidene Fluoride for Flexible Electromechanical Devices: A Review. *Micromachines (Basel)* **11**, (2020).

57. Maji S*, et al.* Self-oriented beta-crystalline phase in the polyvinylidene fluoride ferroelectric and piezo-sensitive ultrathin Langmuir-Schaefer film. *Phys Chem Chem Phys* **17**, 8159-8165 (2015).

58. Mu C, Su Y, Sun M, Chen W, Jiang Z. Fabrication of microporous membranes by a feasible freeze method. *Journal of Membrane Science* **361**, 15-21 (2010).

59. Zhang W, Zaarour B, Zhu L, Huang C, Xu B, Jin X. A comparative study of electrospun polyvinylidene fluoride and poly(vinylidenefluoride-co-trifluoroethylene) fiber webs: Mechanical properties, crystallinity, and piezoelectric properties. *Journal of Engineered Fibers and Fabrics* **15**, (2020).

60. Hu X, You M, Yi N, Zhang X, Xiang Y. Enhanced Piezoelectric Coefficient of PVDF-TrFE Films via In Situ Polarization. *Frontiers in Energy Research* **9**, (2021).

61. Li J*, et al.* Multifunctional Artificial Artery from Direct 3D Printing with Built-In Ferroelectricity and Tissue-Matching Modulus for Real-Time Sensing and Occlusion Monitoring. *Adv Funct Mater* **30**, (2020).

62. Li J, Yang F, Long Y, Dong Y, Wang Y, Wang X. Bulk Ferroelectric Metamaterial with Enhanced Piezoelectric and Biomimetic Mechanical Properties from Additive Manufacturing. *ACS Nano* **15**, 14903-14914 (2021).

63. Baji A, Mai Y-W, Li Q, Liu Y. Nanoscale investigation of ferroelectric properties in electrospun barium titanate/polyvinylidene fluoride composite fibers using piezoresponse force microscopy. *Composites Science and Technology* **71**, 1435-1440 (2011).

64. Kim HS*, et al.* Dominant Role of Young's Modulus for Electric Power Generation in PVDF(-)BaTiO(3) Composite-Based Piezoelectric Nanogenerator. *Nanomaterials (Basel)* **8**, (2018).

65. Han J, Li D, Zhao C, Wang X, Li J, Wu X. Highly Sensitive Impact Sensor Based on PVDF-TrFE/Nano-ZnO Composite Thin Film. *Sensors (Basel)* **19**, (2019).

66. Zhang C, Wei W, Sun H, Zhu Q. Performance enhancements in poly(vinylidene fluoride)-based piezoelectric films prepared by the extrusion-casting process. *Journal of Materials Science: Materials in Electronics* **32**, 21837-21847 (2021).

67. Li W*, et al.* Nanogenerator-based dual-functional and self-powered thin patch loudspeaker or microphone for flexible electronics. *Nat Commun* **8**, 15310 (2017).

68. Zhang Z, Yao C, Yu Y, Hong Z, Zhi M, Wang X. Mesoporous Piezoelectric Polymer Composite Films with Tunable Mechanical Modulus for Harvesting Energy from Liquid Pressure Fluctuation. *Adv Funct Mater* **26**, 6760-6765 (2016).

69. Genchi GG*, et al.* P(VDF-TrFE)/BaTiO3 Nanoparticle Composite Films Mediate Piezoelectric Stimulation and Promote Differentiation of SH-SY5Y Neuroblastoma Cells. *Adv Healthc Mater* **5**, 1808-1820 (2016).

70. Yan W*, et al.* Single fibre enables acoustic fabrics via nanometre-scale vibrations. *Nature* **603**, 616-623 (2022).
