# Supplementary material for: Soft ferroelectret ultrasound receiver for targeted peripheral neuromodulation
Source: Nat Commun. 2023 Dec 16;14:8386. doi: 10.1038/s41467-023-44065-6 (PMC10725454; doi:10.1038/s41467-023-44065-6)
Supplement: Supplementary file 3 — Description of Additional Supplementary Files [file 41467_2023_44065_MOESM3_ESM.pdf]

**Description of Additional Supplementary Files**

**Supplementary Movies**

**Supplementary Movie 1**. High-speed vibrating ferroelectric film responding to US vibrations. The ferroelectret-based NeuroRing acts as an US receiver, converting US vibrations into electrical impulses.

**Supplementary Movie 2.** Ankle flexion motion induced by the US-excited NeuroRing. This phenomenon demonstrates that the electrical pulses generated by the US-excited NeuroRing can effectively activate nerves, suggesting its clinical potential in therapeutic neuromodulation.

**Supplementary Movie 3.** EEG signal fluctuations induced by the US-excited NeuroRing. Once US activates the NeuroRing at the sacral nerve, the EEG signals change and intensify
