# Supplementary material for: Soft ferroelectret ultrasound receiver for targeted peripheral neuromodulation
Source: Nat Commun. 2023 Dec 16;14:8386. doi: 10.1038/s41467-023-44065-6 (PMC10725454; doi:10.1038/s41467-023-44065-6)
Supplement: Supplementary file 7 — Reporting Summary [file 41467_2023_44065_MOESM7_ESM.pdf]

nature portfolio

Corresponding author(s): Zhang-Qi Feng

Last updated by author(s): Nov 19, 2023

# Reporting Summary

Nature Portfolio wishes to improve the reproducibility of the work that we publish. This form provides structure for consistency and transparency in reporting. For further information on Nature Portfolio policies, see our Editorial Policies and the Editorial Policy Checklist.

## Statistics

For all statistical analyses, confirm that the following items are present in the figure legend, table legend, main text, or Methods section.

| n/a | Confirmed | |
|---|---|---|
| ☐ | ☒ | The exact sample size (*n*) for each experimental group/condition, given as a discrete number and unit of measurement |
| ☐ | ☒ | A statement on whether measurements were taken from distinct samples or whether the same sample was measured repeatedly |
| ☐ | ☒ | The statistical test(s) used AND whether they are one- or two-sided<br>*Only common tests should be described solely by name; describe more complex techniques in the Methods section.* |
| ☒ | ☐ | A description of all covariates tested |
| ☒ | ☐ | A description of any assumptions or corrections, such as tests of normality and adjustment for multiple comparisons |
| ☐ | ☒ | A full description of the statistical parameters including central tendency (e.g. means) or other basic estimates (e.g. regression coefficient) AND variation (e.g. standard deviation) or associated estimates of uncertainty (e.g. confidence intervals) |
| ☐ | ☒ | For null hypothesis testing, the test statistic (e.g. *F*, *t*, *r*) with confidence intervals, effect sizes, degrees of freedom and *P* value noted<br>*Give P values as exact values whenever suitable.* |
| ☒ | ☐ | For Bayesian analysis, information on the choice of priors and Markov chain Monte Carlo settings |
| ☒ | ☐ | For hierarchical and complex designs, identification of the appropriate level for tests and full reporting of outcomes |
| ☒ | ☐ | Estimates of effect sizes (e.g. Cohen's *d*, Pearson's *r*), indicating how they were calculated |

*Our web collection on statistics for biologists contains articles on many of the points above.*

## Software and code

Policy information about availability of computer code

| | |
|---|---|
| Data collection | Piezoelectric voltage data were collected with oscilloscope (UTD2102CAL) with a voltage probe (Tektronix P5100A) with 40 MΩ input impedance; and mechanical tests were collected with computer servo tensile tester (HD-B609B-S). Cell images and all tissue slides were collected with fluorescence microscopy (SP8, Leica); EMG and EEG data were collected with a multi-channel physiological signal acquisition and recording instrument (RM6240). No commercial, open-source, nor custom code was used for data collection. |
| Data analysis | Piezoelectric voltage data and mechanical test data were analysed by using OriginalPro 2016 b9.3.226 (64-bit) and Microsoft Excel 2016 (version 1807, 10325.20082). Cell images and immunofluorescence data were analysed by using ImageJ (Fiji), OriginalPro 2016 b9.3.226 (64-bit) and Microsoft Excel 2016 (version 1807, 10325.20082). EMG and EEG data were analysed by using custom code in Matlab R2017a, OriginalPro 2016 b9.3.226 (64-bit) and Microsoft Excel 2016 (version 1807, 10325.20082). P value was calculated by software IBM SPSS Statistics. The projection X-ray images were reconstructed into 3D images and processed using software Dragonfly (Object Research System). |

For manuscripts utilizing custom algorithms or software that are central to the research but not yet described in published literature, software must be made available to editors and reviewers. We strongly encourage code deposition in a community repository (e.g. GitHub). See the Nature Portfolio guidelines for submitting code & software for further information.

## Data

Policy information about availability of data

All manuscripts must include a data availability statement. This statement should provide the following information, where applicable:

- Accession codes, unique identifiers, or web links for publicly available datasets
- A description of any restrictions on data availability
- For clinical datasets or third party data, please ensure that the statement adheres to our policy

The authors declare that all data supporting the findings of this study are available within the paper and its Supplementary Information.

## Research involving human participants, their data, or biological material

Policy information about studies with human participants or human data. See also policy information about sex, gender (identity/presentation), and sexual orientation and race, ethnicity and racism.

| | |
|---|---|
| Reporting on sex and gender | The study did not involve human research participants. |
| Reporting on race, ethnicity, or other socially relevant groupings | This study did not include human samples. |
| Population characteristics | This study did not include human samples. |
| Recruitment | This study did not include human samples. |
| Ethics oversight | This study did not include human samples. |

Note that full information on the approval of the study protocol must also be provided in the manuscript.

# Field-specific reporting

Please select the one below that is the best fit for your research. If you are not sure, read the appropriate sections before making your selection.

☒ Life sciences ☐ Behavioural & social sciences ☐ Ecological, evolutionary & environmental sciences

For a reference copy of the document with all sections, see nature.com/documents/nr-reporting-summary-flat.pdf

# Life sciences study design

All studies must disclose on these points even when the disclosure is negative.

| | |
|---|---|
| Sample size | Sample size was not calculated beforehand. Sample size was determined by the number of biological and technical replicates necessary to convince us that the effect was real. The number of biological replicates we aimed for was at least 3, with several technical replicates in each sample. |
| Data exclusions | No data were excluded from the analyses. |
| Replication | All experimental findings, including material fabrication and characterization, and animal experiments were reliably reproduced. |
| Randomization | Experimental groups were formed based on what was being tested with random selections. The same type of animals were used for all experiments. |
| Blinding | The investigators were not blinded. |

# Reporting for specific materials, systems and methods

We require information from authors about some types of materials, experimental systems and methods used in many studies. Here, indicate whether each material, system or method listed is relevant to your study. If you are not sure if a list item applies to your research, read the appropriate section before selecting a response.

## Materials & experimental systems

| n/a | Involved in the study |
|---|---|
| ☐ | ☒ Antibodies |
| ☐ | ☒ Eukaryotic cell lines |
| ☒ | ☐ Palaeontology and archaeology |
| ☐ | ☒ Animals and other organisms |
| ☒ | ☐ Clinical data |
| ☒ | ☐ Dual use research of concern |
| ☒ | ☐ Plants |

## Methods

| n/a | Involved in the study |
|---|---|
| ☒ | ☐ ChIP-seq |
| ☒ | ☐ Flow cytometry |
| ☒ | ☐ MRI-based neuroimaging |

# Antibodies

| | |
|---|---|
| Antibodies used | Antibodies used in this work include anti-Tuj-1 (Abcam); anti-NF200 (Abcam); anti-TNF-α (Abcam); anti-CD11b (Abcam); anti-CD11c (Abcam); anti-CD206 (Abcam); anti-F/480 (Abcam); anti-HO-1 (Abcam); anti-Caspase1 (Abcam); anti-IL6 (Abcam) and anti-IL10 (Abcam). |
| Validation | Validation of each antibody was done standard information offered by the supplier. |

# Eukaryotic cell lines

Policy information about cell lines and Sex and Gender in Research

| | |
|---|---|
| Cell line source(s) | SH-SY5Y cells (Human Neuroblastoma Cell Line) were obtained from the Cell Bank, Shanghai Institutes for Biological Sciences, Chinese Academy of Sciences. |
| Authentication | Identity of the cell lines were frequently checked by their morphological features but have not been authenticated by the short tandem repeat profiling. |
| Mycoplasma contamination | The cell line was tested for mycoplasma contamination. No mycoplasma contamination was found. |
| Commonly misidentified lines (See ICLAC register) | No commonly misidentified cell lines are used in this study. |

# Animals and other research organisms

Policy information about studies involving animals; ARRIVE guidelines recommended for reporting animal research, and Sex and Gender in Research

| | |
|---|---|
| Laboratory animals | Male Sprague-Dawley rats (150 to 200 g) were used. |
| Wild animals | The study did not involve wild animals. |
| Reporting on sex | The study did not involve sex-based analyses |
| Field-collected samples | The study did not involve field-collected samples |
| Ethics oversight | All procedures involving implantation and ultrasound stimulation were handled in accordance with Institutional Animal Care and Use Committee approval protocol of the Animal Care Center at the Nanjing Jinling Hospital. |

Note that full information on the approval of the study protocol must also be provided in the manuscript.
